# Transcriptomes of antigen presenting cells in human thymus

**DOI:** 10.1371/journal.pone.0218858

**Published:** 2019-07-01

**Authors:** Ingvild S. M. Gabrielsen, Hanna Helgeland, Helle Akselsen, Hans Christian D. Aass, Arvind Y. M. Sundaram, Isaac V. Snowhite, Alberto Pugliese, Siri T. Flåm, Benedicte A. Lie

**Affiliations:** 1 Department of Medical Genetics, University of Oslo and Oslo University Hospital, Oslo, Norway; 2 K. G. Jebsen Inflammation Research Centre, University of Oslo, Oslo, Norway; 3 Institute of Clinical Medicine, University of Oslo, Oslo, Norway; 4 Department of Medical Biochemistry, Oslo University Hospital, Oslo, Norway; 5 Diabetes Research Institute, Miller School of Medicine, University of Miami, Miami, Florida, United States of America; 6 Department of Medicine, Division of Endocrinology, Diabetes and Metabolism, Microbiology and Immunology, Miller School of Medicine, University of Miami, Miami, Florida, United States of America; University of Alberta, CANADA

## Abstract

Antigen presenting cells (APCs) in the thymus play an essential role in the establishment of central tolerance, i.e. the generation of a repertoire of functional and self-tolerant T cells to prevent autoimmunity. In this study, we have compared the transcriptomes of four primary APCs from human thymus (mTECs, CD19^+^ B cells, CD141^+^ and CD123^+^ DCs). We investigated a set of genes including the HLA genes, genes encoding transcriptional regulators and finally, tissue-enriched genes, i.e, genes with a five-fold higher expression in a particular human tissue. We show that thymic CD141^+^ DCs express the highest levels of all classical HLA genes and 67% (14/21) of the HLA class I and II pathway genes investigated in this study. CD141^+^ DCs also expressed the highest levels of the transcriptional regulator *DEAF1*, whereas *AIRE* and *FEZF2* expression were mainly found in primary human mTECs. We found expression of “tissue enriched genes” from the Human Protein Atlas (HPA) in all four APC types, but the mTECs were clearly dominating in the number of uniquely expressed tissue enriched genes (20% in mTECs, 7% in CD19^+^ B cells, 4% in CD123^+^ DCs and 2% in CD141^+^ DCs). The tissue enriched genes also overlapped with reported human autoantigens. This is, to our knowledge, the first study that performs RNA sequencing of mTECs, CD19^+^ B cells, CD141^+^ and CD123^+^ DCs isolated from the same individuals and provides insight into the transcriptomes of these human thymic APCs.

## Introduction

Antigen presenting cells (APCs) in the thymus are essential for the establishment of central tolerance. By presenting self-peptides to the developing thymocytes, they contribute in the critical process of selecting thymocytes with functionally competent T-cell receptors tolerant to the body’s tissues and organs. Due to comprehensive studies performed the last decades, mainly in mice, extensive knowledge about how thymic APCs mediate central tolerance has been obtained [1–9]. Here, we focus on four different types of APCs; CD141^+^ and CD123^+^ dendritic cells (DCs), CD19^+^ B cells and medullary thymic epithelial cells (mTECs).

The most widely studied thymic APC, the mTEC, is a specialized cell type that transcribes a large number of tissue-specific genes [2]. Expression of these genes encoding tissue-restricted antigens (TRAs) contrasts with the tight spatio-temporal control of gene expression in peripheral tissues during pre- and post-natal development and has been termed “promiscuous gene expression” (PGE) [10–15]. A given TRA is lowly expressed and only expressed in a minority of mTECs (1–3%) at any given time [1]. Approximately 40% of the TRAs [16] are under the transcriptional control of the autoimmune regulator (Aire). This regulator protein is crucial for the establishment of central tolerance, and loss-of-function mutations in *AIRE* cause a recessive autoimmune syndrome termed autoimmune polyendocrinopathy-candidiasis-ectodermal dystrophy (APECED) in humans [17]. Takaba et al. recently reported a second regulator in mTECs, the Fez family zinc-finger 2 (Fezf2), which mediates the expression of Aire-independent TRAs [3]. Furthermore, the major subset of DCs found in the thymus belong to the conventional DC (cDC) lineage, and can be classified as CD8α^+^SIRPα^-^ cDCs in mice [1] or CD141^+^ in humans [18, 19]. CD8α^+^SIRPα^-^ cDCs originate intrathymically and can present TRAs that have been transferred by mTECs [20]. CD123^+^ plasmacytoid DCs are also present, however, these cells acquire antigens from the blood stream before migrating into the thymus where they present them to the developing T-lymphocytes [4, 5]. In this way, they may contribute with self-peptides that are not already included in the spectrum of TRAs promiscuously expressed by mTECs [21]. Whether thymic DCs also transcribe and express their own TRAs is not clear. Finally, thymic B cells are also capable of presenting peptides to the developing thymocytes and induce negative selection [6–8]. The origin of thymic B cells is not fully understood, as both development from intrathymic progenitors [6] and migration from the peripheral circulation [8] have been suggested. In thymic B cells, a subset expressing Aire and Aire-dependent TRAs have been reported in mice [8, 22] and recently, expression of *AIRE* and a few TRA genes were also detected in human thymic B cells [23].

Aire and Fezf2 are not the only known transcriptional regulators of TRA expression. The deformed autoregulatory factor 1 (Deaf1) controls the expression of approximately 600 genes in the pancreatic lymph nodes [24] where around half of the genes were upregulated and half were downregulated in Deaf1knockout. Among the downregulated genes, almost three quarters were encoding potential peripheral tissue-antigens. Deaf1 therefore acts as a potential transcriptional regulator of TRAs, and to date, *DEAF1* expression in human thymic APC has yet not been examined.

To our knowledge, RNA sequencing of human thymic APCs has only been performed in B cells [23]. In this study, we have performed high-throughput RNA sequencing to compare the transcriptomes of four different, primary APCs (mTECs, CD141^+^ DCs, CD123^+^ DCs and CD19^+^ B cells) from human thymus, and investigated a set of genes including the HLA genes, genes encoding transcriptional regulators and tissue-enriched genes.

## Results

### Purification of primary thymic APC

**Fig 1:**
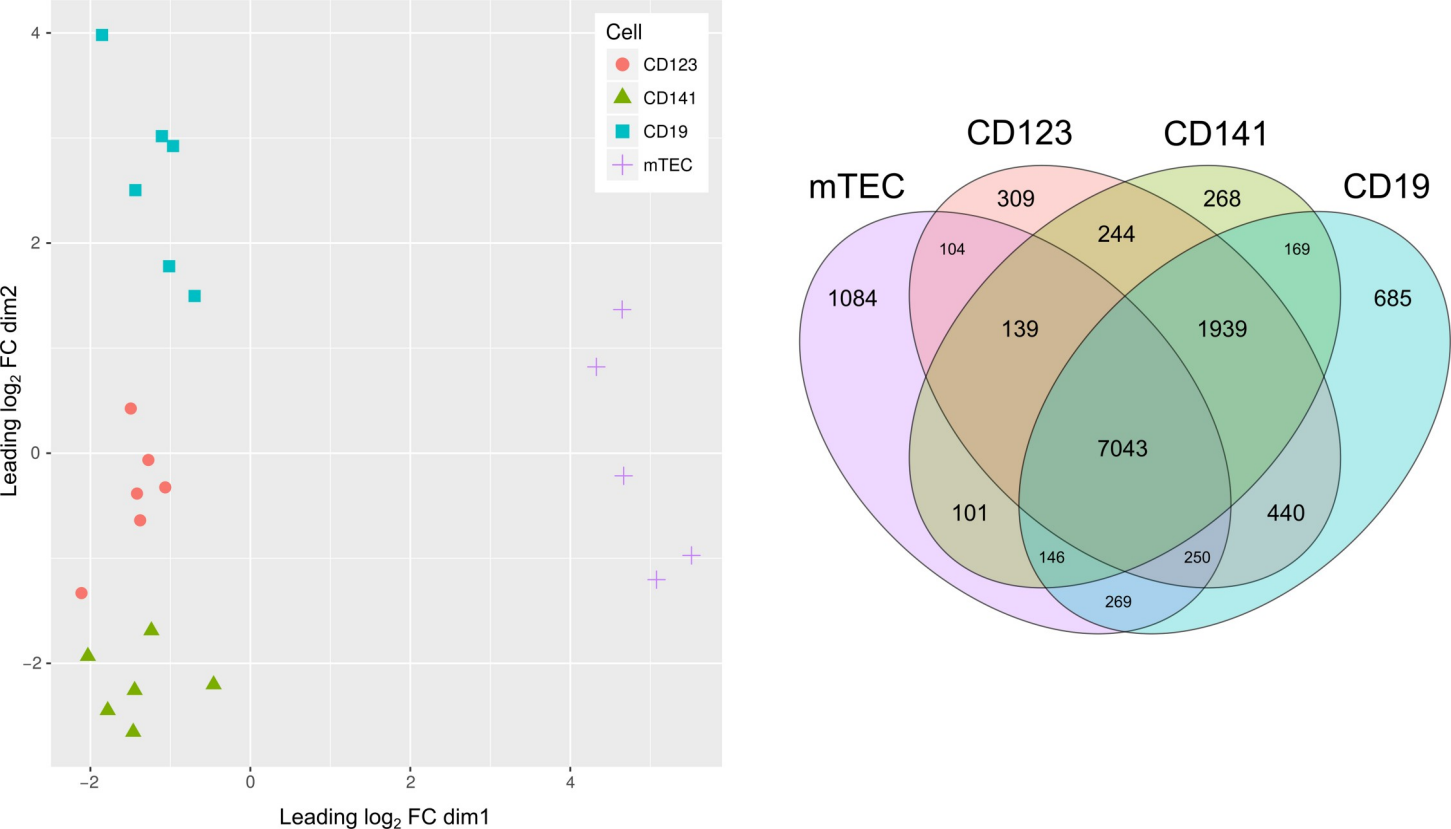
Thymic APC clustering and their number of expressed genes. (A) MDS plot displaying the leading log_2_-fold-change distance between the thymic APC samples (B) Number of uniquely and commonly expressed genes (FPKM > 1) between the four thymic APCs.

We isolated four different cell populations (mTECs, CD141^+^ DCs, CD123^+^ DCs and CD19^+^ B cells) from six human thymic samples, where one biological replicate was removed from the mTECs due to contamination. After RNA sequencing and trimming of the data, the average library size was 59.5 ± 8.3 million mapped paired reads for the APC samples (S1 Table). The final APC dataset comprised 15245 Ensembl genes, where transcript levels were quantified in Fragments per Kilobase of transcript per Million mapped reads (FPKM). A multi-dimensional scaling (MDS) plot (Fig 1A) of leading log_2_-fold-change showed that the samples from the same thymic APC subtype tended to group together in distinct clusters, indicating that the variation between the four cell populations is higher than within the four populations. To validate the purity of our cell populations, we investigated the expression of indicator genes (S1 and S2 Figs) previously established in the human thymic cell types (S2 Table). Taken together, we regarded these cell populations as sufficiently pure to pursue comparative gene expression analyses.

### Genes differentially expressed and spliced between the thymic APCs

First, we investigated how genes with expression levels FPKM > 1 were distributed between the thymic APCs (Fig 1B). We found that 46% (n = 7043) of the 15245 genes present in our dataset were expressed (FPKM >1) in all four thymic APC cell types, while 13% of the genes in the dataset had FPKM levels below 1 in all APC populations. The DCs (i.e. the CD123+ and/or the CD141+) shared 66% (n = 7043 + 1939 + 169 + 146 + 250 + 440) of the expressed genes with B cells whereas only 51% (n = 7043 + 139 + 104 + 250 + 146 + 101) were shared between the DCs and the mTECs. B cells and mTECs also shared 51% (n = 7043 + 269 + 146 + 250) of the expressed genes in the dataset. The percentage of all expressed genes that were detected uniquely was 7% in mTECs (n = 1084), 4% (n = 685) in CD19^+^ B cells, 2% (n = 309) in CD123^+^ DCs, and finally 2% (n = 268) in CD141^+^ DCs. A list of the unique and commonly expressed genes (FPKM > 1) between the four thymic APCs has been provided in S3 Table. We continued by exploring significantly differentially expressed (DE) and differentially spliced (DS) genes between the thymic APCs (Table 1 and S3 Fig). The largest number of significantly DE and DS genes was seen between mTECs and non-epithelial APCs (2787–3093 DE genes and 339–372 DS genes). Fewer DE and DS genes were observed when the CD19^+^ B cells were compared to the DC subsets (284–769 DE genes and 26–47 DS genes) and between the CD123^+^ and the CD141^+^ DCs (139 DE genes and 28 DS genes). A list of the significantly DE genes has been provided in S4 Table. Furthermore, we performed a gene ontology (GO) enrichment analysis of the significant DE genes (log_2_ FC > 1, FDR < 0.05) pairwise between the APCs (S4–S9 Figs). The most significant GO term in CD141^+^, CD123^+^ and CD19^+^ compared to mTECs was “immune system process”. Conversely, the most significant GO term in mTECs compared to CD141^+^, CD123^+^ and CD19^+^ B cells was “anatomical structure development”. Interestingly, we also observed GO term enrichment branching down to more specific terms in the mTECs, such as “regulation of nervous system development” and “muscle system process”.

**Table 1:** Pairwise comparison of A. differentially expressed (DE) genes (log_2_ fold change >1; FDR P-value <0.05) and B. differentially spliced genes (FPKM >1; FDR P-value < 0.05) in the four thymic APCs.

APC pairs		A. DE genes			B. DS genes
Cell type 1	Cell type 2	Number of genes higher expressed in cell type 1	Number of genes higher expressed in cell type 2	Total number of DE genes	Total number of genes with differential exon usage
mTECs	CD141^+^	1527	1566	3093	372
mTECs	CD123^+^	1230	1653	2883	355
mTECs	CD19^+^	1165	1622	2787	339
CD19^+^	CD141^+^	434	335	769	47
CD19^+^	CD123^+^	75	209	284	26
CD141^+^	CD123^+^	33	106	139	28

### HLA and genes involved in the HLA class I and II pathways

**Fig 2:**
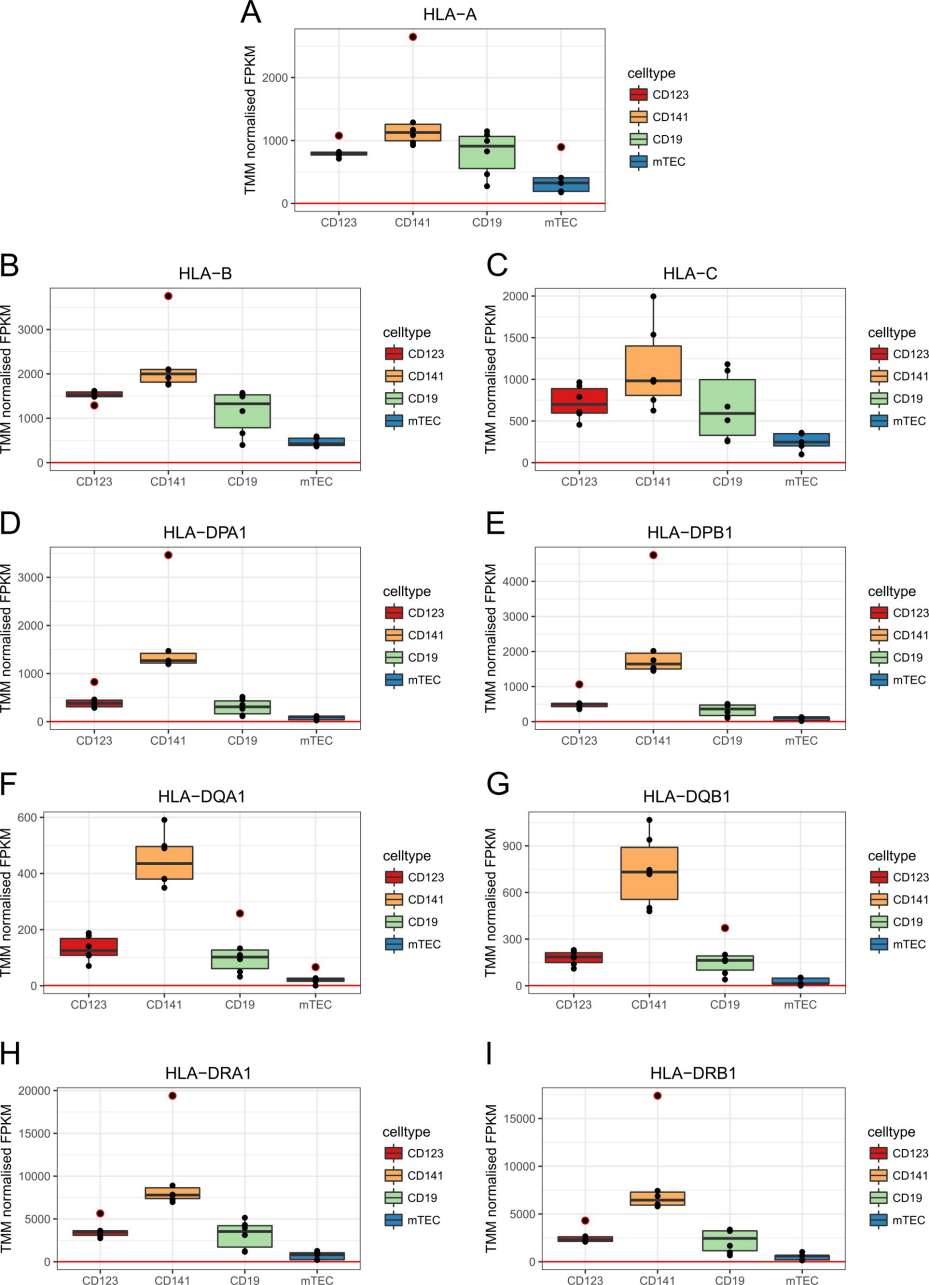
Expression levels of HLA genes in the individual APCs. Boxplots represent the median and quartiles of the relative RNA expression levels as normalized FPKM. The X-axis shows the individual thymic APCs and the Y-axis shows the TMM normalized FPKM. An expression level minimum has been set at FPKM = 1 (red line). Black dots represent the individual biological replicates. Black dots encircled in red are outliers. (A) *HLA-A* (B) *HLA-B* (C) *HLA-C* (D) *HLA-DPA1* (E) *HLA-DPB1* (F) *HLA-DQA1* (G) *HLA-DQB1* (H) *HLA-DRA1* (I) *HLA-DRB1*. The expression of alpha and beta chain genes for HLA class II molecules was consistently following each other.

We next analyzed the expression levels of the classical HLA genes and the genes involved in the HLA class I and class II antigen presentation pathways [25]. For all the classical HLA genes, the CD141^+^ DCs expressed the highest levels (Fig 2).

The class I genes *HLA-B* and *HLA-C* displayed the highest and lowest expression levels in all APCs, respectively. The class II genes *HLA-DRA1* and *HLA-DRB1* obtained the highest expression levels, whereas the lowest expression levels varied between *HLA-DQA1* and *HLA-DQB1*. The classical HLA genes significantly DE (FDR < 0.05) between the thymic APCs have been listed in S5 Table. Among the genes involved in the HLA class I antigen presentation pathway (S10 Fig), we observed that the CD141^+^ DCs also expressed the highest levels of *B2M* (β2-microglobulin), *CALR* (calreticulin), *ERAP2* (endoplasmic reticulum aminopeptidase 2), *PDIA3* (protein disulfide isomerase family A member 3, also known as ERp57), *PSMB8* (immunoproteaosome subunit β5i), *TAP1*, *TAP2* and *TAPBP* (the TAP transporters and tapasin, respectively). *CANX* (calnexin) and *ERAP1* was highest in the CD123+ DCs, whereas *PSMB5* (house-keeping proteaosome subunit β5) was highest in mTECs. Among the genes involved in the HLA class II antigen presentation pathway (S11 Fig), the CD141^+^ DCs expressed the highest levels of *CD74* (invariant chain), *HLA-DMA*, *HLA-DMB*, *HLA-DOA*, *HLA-DOB* (the heterodimeric glycoproteins DM and DO) and *IFI30* (the lysosomal thiol reductase, also known as GILT). The genes *CTSB* and *CTSS*, encoding cathepsins B and S, and *LGMN*, encoding the asparaginyl endopeptidase (all three are involved in proteolysis of antigens in the endosomal and lysosomal compartments before HLA class II loading) showed the highest expression in CD123^+^ DCs, while *CTSF* (cathepsins F) was highest in mTECs. Genes involved in the HLA class I and II antigen presentation pathway that were significantly DE among the APC pairs are reported in S6 Table.

### Transcriptional regulator genes

**Fig 3:**
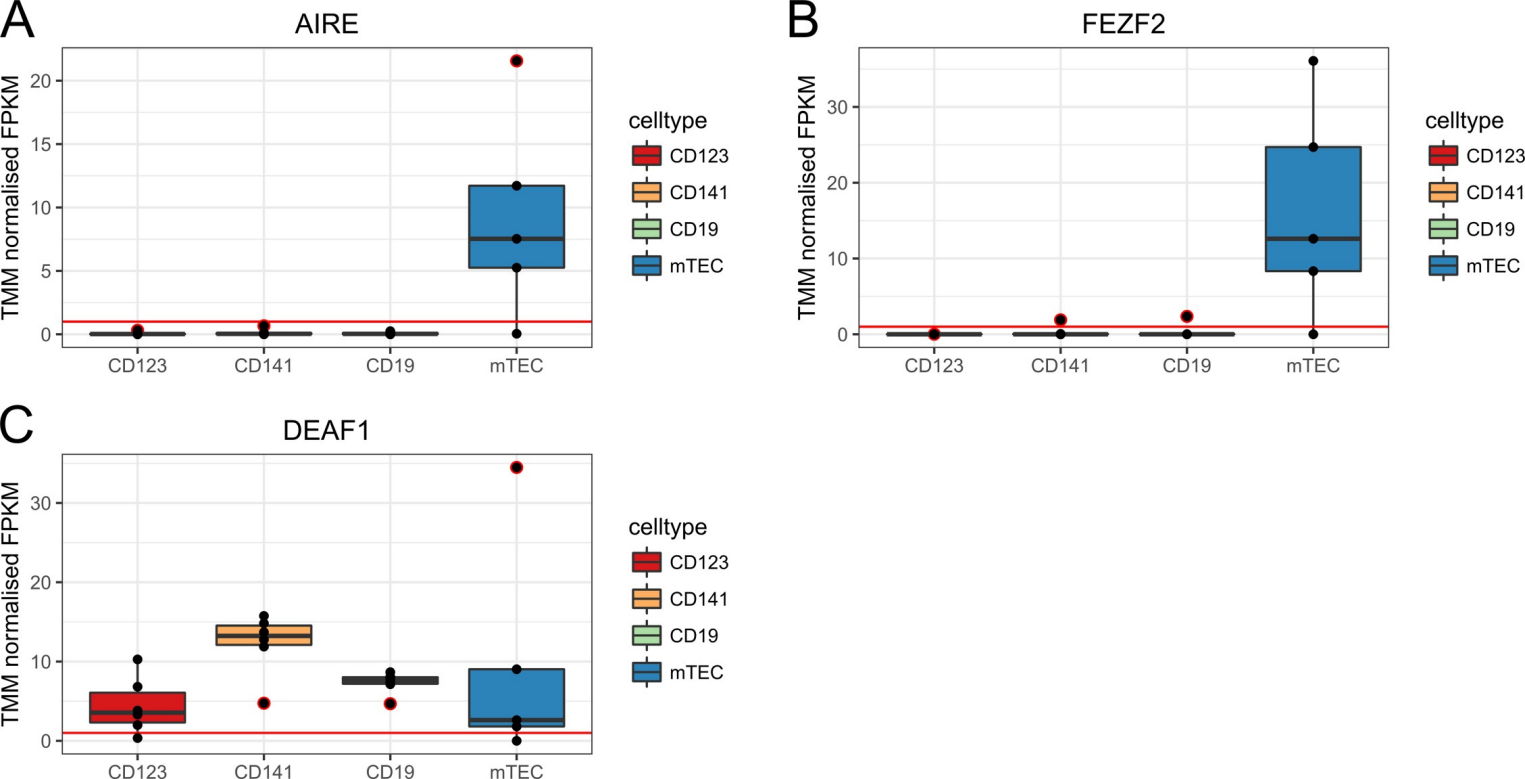
Expression levels of the three transcriptional regulators. (A) *AIRE* (B) *FEZF2* and (C) *DEAF1*. Boxplots represent the median and quartiles of the relative RNA expression levels as normalized FPKM. The X-axis shows the individual thymic APCs and the Y-axis shows the TMM normalized FPKM. An expression level minimum has been set at FPKM = 1 (red line). Black dots represent the individual biological replicates. Black dots encircled in red are outliers.

We continued by investigating the expression level of three transcriptional regulator genes, *AIRE*, *FEZF2* and *DEAF1* (Fig 3) in the human thymic APCs. The mTECs were the only APC type that showed a median expression level of *AIRE* and *FEZF2* above FPKM = 1. On the contrary, *DEAF1* was expressed in all APCs, but the highest levels were clearly detected in the CD141^+^ DCs.

### Tissue enriched genes in the thymic APCs

**Fig 4:**
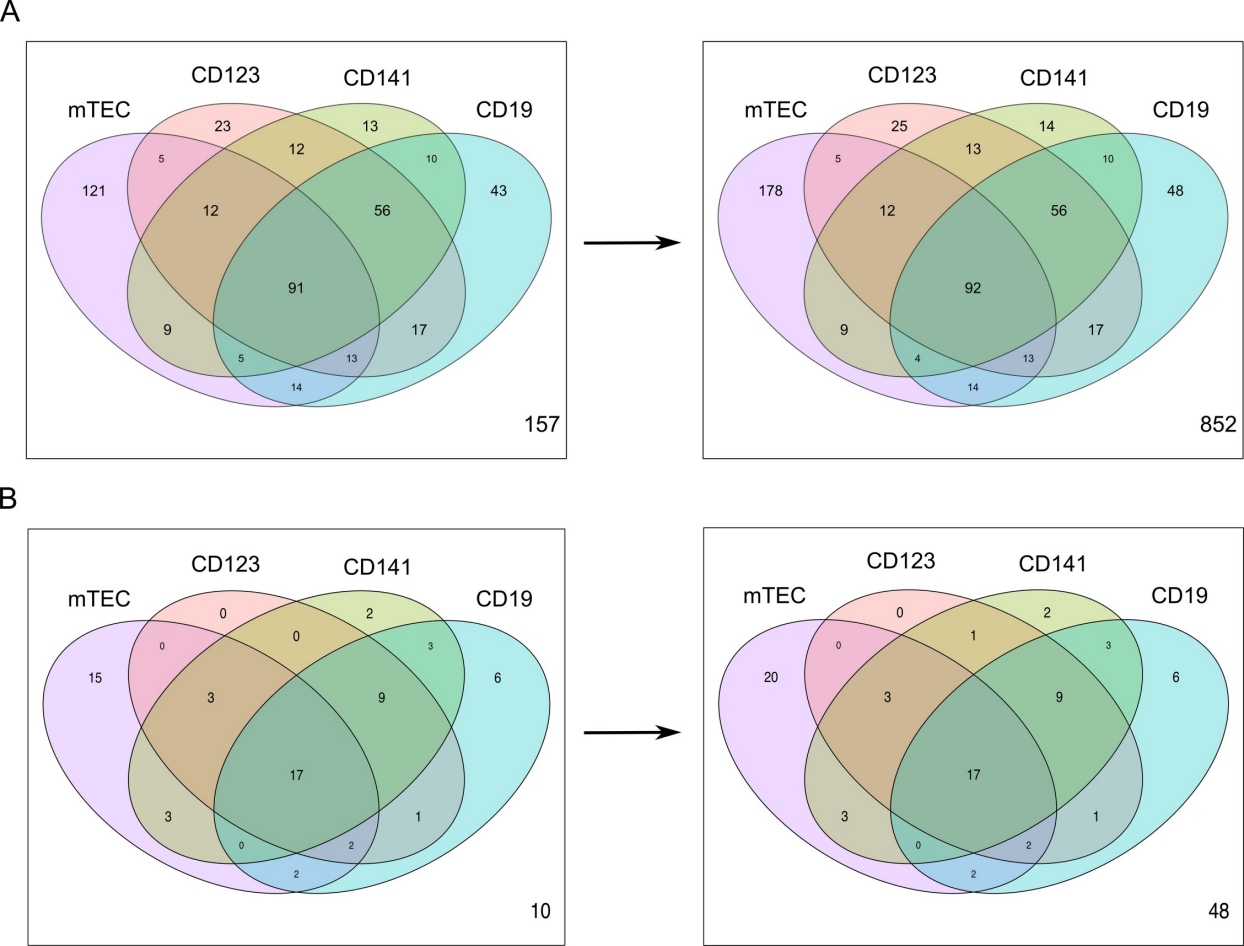
Uniquely and commonly expressed tissue enriched genes in the thymic APCs. Venn diagram showing the number of expressed (FPKM > 1) (A) tissue enriched genes and (B) tissue enriched genes that overlap with genes encoding human autoantigens reported in the Immune Epitope Database. The arrow indicates that the number of biological replicates that a gene needed to be expressed in to be included in the dataset changed from five to one. The number down in the right corner displays the number of genes with expression levels FPKM < 1.

Furthermore, we wanted to explore to what extent genes encoding TRAs were expressed in the thymic APCs. However, this turned out to be a more complicated task than anticipated, as large-scale projects such as the Human protein atlas (HPA) have shown that many “tissue-specific” proteins from the literature are in fact expressed in several tissues [26]. We therefore used the list of “tissue enriched” genes from the HPA, (i.e. genes where mRNA levels in one tissue type are at least five times the maximum levels of all other tissues analyzed) and investigated the expression level of these genes in the thymic APCs. A total of 601 tissue enriched genes were present in our APC dataset, and the percentage of all expressed genes (FPKM >1) that were detected uniquely was 20% in mTECs (n = 121), 7% (n = 43) in CD19^+^ B cells, 4% (n = 23) in CD123^+^ DCs and 2% (n = 13) in CD141^+^ DCs (Fig 4A). A total of 15% (n = 91) of the tissue enriched genes were expressed in all four APCs while 26% (n = 157) of the genes had FPKM levels < 1.

It has been reported in the literature that each individual TRA is only expressed in a minority of the mTECs (1–3%) at any given time [1]. We therefore questioned whether our strict edgeR data threshold (generally set to avoid false positives), stating that genes need to be present in five biological replicates to be included in the APC dataset, restricted the number of tissue enriched genes in our analysis. Therefore, we reanalyzed the data with a lower threshold, where genes only needed to be present in one biological replicate to be included in the dataset (Fig 4A and S7 Table). The number of unique tissue enriched genes then increased remarkably in the mTECs (from 121 to 178, Δ = 57) compared to the other APCs (Δ = 5 in CD19^+^ B cells, Δ = 2 in CD123^+^ DCs and 1 in CD141^+^ DCs). The percentage of tissue enriched genes shared between the four APCs was 7%, however, the number of genes with FPKM < 1 increased from 26% to 63% (n = 852), indicating that quite a lot of the tissue enriched genes are very lowly expressed. We therefore plotted the number of tissue enriched genes in the four APCs across FPKM thresholds ranging from 0.5–11 (S12 Fig). We then observed that that the mTECs expressed the highest number of tissue enriched genes, regardless of the FPKM threshold. Finally, we investigated whether there was any overlap between the tissue enriched genes and genes encoding human autoantigens (Fig 4B) from the Immune Epitope database (see materials and methods). All the APCs expressed tissue enriched genes overlapping with autoantigen genes (S8 Table).

### Autoimmune expression quantitative trait loci genes in the APCs

We have previously reported autoimmune disease associated expression quantitative trait loci (eQTL) in whole human thymus [27, 28]. As the thymic tissue is composed of 98% thymocytes and only 2% APCs [29], it is conceivable that the expression of these eQTL genes (eGenes) originated from the developing T cells. Nonetheless, we questioned whether five of the eGenes (*FCRL3*, *ERAP2*, *RNASET2*, *SIRPG* and *SYS1)* were expressed in the thymic APCs, and also included 10 suggested eGenes with P-values < 7.4 x 10^−4^ that did not reach the significance threshold in the eQTL study [27]. We observed that all the eGenes were expressed (FPKM > 1) in at least one of the APCs (S13 and S14 Figs). While *IP6K1*, *PARK7*, *SYS1* and *TROVE2* were expressed (FPKM > 1) in all four cell types, *SLC16A14* was the only eGene with a median FPKM expression level above 1 uniquely in one of the APC subsets (the CD141^+^ DCs). However, we could detect variable levels of *SLC16A14* among the biological replicates in the mTECs and in the CD123+ DCs, indicating that this gene is not strictly CD141+ specific.

## Discussion

In this study, we compare the transcriptome data of four human thymic APC subsets obtained from children undergoing heart surgery at very young age. We show that the thymic CD141+ DC is the most active APC in terms of expressing HLA and HLA pathway genes. Our data also supports that mTECs express *AIRE*, *FEZF2* and a high variety of tissue enriched genes. This insight contributes to the field of transcriptomics and the gene lists generated in this study provide a rich resource to the scientific community. This is, to our knowledge, the first study that performs RNA sequencing of mTECs, CD141^+^ DCs, CD123^+^ DCs and CD19^+^ B cells isolated from the same individuals.

The finding that nearly half (46%) of all the genes in our dataset were expressed (FPKM > 1) in all four thymic APCs suggests that these gene products are needed for common house-keeping functions, energy generation, cell growth and basic metabolism. We have also seen from the analysis (Fig 2 and S10 Fig) that the four thymic APCs share certain genes involved in antigen processing and HLA presentation. Furthermore, we observed that a larger number of genes were shared between the DCs and the B cells, whereas the mTECs expressed more unique genes. Also, the largest number of both DE and DS genes was found between mTECs and the non-epithelial APCs. This could partly reflect the fact that mTECs derive from a non-hematopoietic cell lineage, whereas both B cells and DCs derives from hematopoietic cell precursors. When we compared CD123^+^, CD141^+^ and CD19^+^ cells to mTECs, we found that these hematopoietic cells expressed more genes involved in immune system processes, reflecting their function as immune cells. Conversely, mTECs seemed to express more genes involved in “anatomical structure development”, reflecting their function as epithelial cells. The GO terms “regulation of nervous system development” and “muscle system process” also turned up, which could be due to the expression of tissue enriched genes. However, it could also be caused by the presence of rare epithelial cells with an expression phenotype resembling that of cells from muscle and neurons [29].

One interesting finding was that the human CD141^+^ DCs expressed (FPKM > 1) the highest levels of all classical HLA genes and 67% of the HLA class I and II antigen presentation pathway genes investigated in this study. The high HLA expression in CD141^+^ DCs, and simultaneously low expression of tissue enriched genes compared to the other APCs, could indicate that this cell type focuses on the presentation of extracellular peptides derived from the thymic environment. Conversely, primary mTECs expressed the lowest levels of HLA compared to the other thymic APCs. Among the HLA pathway genes, where CD141^+^ DCs expressed the highest levels, mTECs consequently expressed the lowest levels, whereas thymic B cells and CD123^+^ DCs had more similar levels. It should be noted that surface protein expression levels cannot be directly deduced from the mRNA levels, due to variations in e.g. transcriptional and translational rates, and/or mRNA and protein stabilities [30]. However, former studies [30, 31] have shown a moderate correlation between mRNA and protein levels which was better than previously thought. It is therefore conceivable that the HLA protein level reflects, to a certain degree, the mRNA level in the thymic APCs.

Consistent with the literature, we found *AIRE* and *FEZF2* expression levels in human mTECs. In the thymic B cells, *AIRE* was expressed at extremely low levels (FPKM = 0.03) compared to mTECs. However, as only 5% of human thymic B cells are AIRE-positive [23], this could explain the low expression levels. We also discovered that *DEAF1* was expressed in all four APCs, with the highest expression levels in CD141^+^ DCs. As Deaf1 is involved in regulating certain genes encoding peripheral tissue antigens in peripheral lymphoid tissues [24] and the thymic APCs are presenters of antigens from the periphery, it would be interesting to further investigate the role of this transcription factor in the thymic APCs.

Furthermore, we found tissue enriched genes in all four thymic APCs, but when we only considered the uniquely expressed tissue enriched genes in these cell types, the mTECs clearly expressed the largest number, especially when we lowered the data threshold. Additionally, several of the expressed tissue enriched genes in all the APCs overlapped with reported human autoantigen genes from the Immune Epitope Database. However, it still remains uncertain to what extent these low-level transcripts are actually translated and presented on the APC surface [32]. More studies concerning the HLA-peptide repertoire in APCs are needed to confirm which tissue enriched peptides that are presented to the developing thymocytes in human thymus, and as stated by others [32, 33], these types of experiments are currently limited by available technology.

Lastly, even though thymocytes are the most abundant cell type in whole thymic tissue, previously reported thymic autoimmune disease associated eGenes were clearly expressed in the thymic APCs. This suggests that gene expression levels might be influenced by risk variants in the thymic APCs. None of the eGenes were clearly cell type-specific. However, the number of eGenes was limited, as the study from where they were obtained [27] was underpowered due to the low number of thymi (n = 42). In the future, larger studies including more thymic tissue samples will most likely reveal more autoimmune disease associated eQTLs, which further encourages to search for autoimmune disease related eQTLs in individual thymic APCs.

In this study, we used five to six biological replicates for each APC type, as it has been reported that the number of DE genes increases with the number of biological replicates (n = 2–6) [34]. This has given strength to our study regarding improved accuracy for log_2_ FC estimates. Furthermore, Liu et al. also reports that, for DE studies, sequencing more than 10 million reads per sample gives diminishing returns compared with adding replication [34]. The majority of the APC samples (22 of 23) comprised between 17 and 37 million paired reads in their libraries (see S1 Table), indicating sufficient sequencing depth for differential expression analysis. One mTEC sample only had 7,219,276 paired reads, but this sample still clustered together with the other mTECs in the MDS plot and was therefore kept in the analyses. Sequencing deeply is also advantageous when analyzing differential expression of exons [34].

To conclude, this study provides data on the transcriptomes of four human thymic APCs, as well as insight into the expression profiles of genes important for the APCs and genes associated with risk for autoimmune diseases.

## Materials and methods

The project is approved by the Regional Ethics Committee (REC) South-East, the Norwegian Social Science Data Service, and the Norwegian Directorate of Health.

### Sample material

Human thymus tissue was obtained from six children undergoing cardiac surgery. All six children were boys within an age range of 24 days– 16 months. None of the patients had any known syndromes. This project was approved by the regional ethical committee and written informed consent was given by all parents. All tissue samples were made anonymous.

### Thymus dissociation

A half thymus (~10 g) was collected each time and immediately washed twice in PBS (Gibco, Thermo Fischer #14190–094, MA, USA) and then stored in a medium consisting of 90% RPMI (Sigma-Aldrich # R7509, MO, USA) and 10% heat inactivated FCS (PAAlab #15–102, Pasching, Austria) for 30 min. The thymic tissue was then divided and treated in two C-tubes (5mg in each) with Collagenase D (Roche Life Science #11088858001, Basel, Switzerland) three times and Liberase (Roche Life Science #05401119001) [35] twice on a gentle MACS Octo Dissociator (Miltenyi Biotec # 130-096-427, Bergisch Gladbach, Germany) to completely dissolve the tissue. The first C-tube was intended for TEC isolation and was treated at 37°C [29], while the second C-tube, intended for B cells and DC isolation, was treated at 20°C [36]. After each dissociation, the supernatants from each tube was filtered and pooled to final, respective cell suspensions, before the cells were counted.

### Isolation of thymic APCs

Four different cell types (mTECs, CD19^+^ B cells, CD123^+^ and CD141^+^ DCs) were isolated from each thymus. After counting the cells in the two total cell suspensions, OptiPrep Density Gradient Medium (Axis Shield, Alere, Oslo, Norway) was used to separate the light density APC cells from the T-lymphocytes. After centrifugation, the layer with the purified APCs treated at 37°C was transferred to one tube, while the layer of APCs treated at 20°C were divided and transferred to two new tubes. The first tube (37°C) was first depleted for CD45^+^ positive cells with the EasySep Human CD45 Depletion kit (STEMCELL Technologies #18259) before TECs were EpCAM-positively selected with CELLection Epithelial Enrich (Thermo Fischer #16203). cTECs were further separated from the mTECs by using anti-CDR2 [37]-biotin and employing EasySep biotin selection kit (STEMCELL Technologies #18553). The cTECs were not used in this study. The second tube (20°C) was used for isolating CD123^+^ and CD141^+^ DCs. These cell types were separated on an autoMACS Pro Separator (MACS Miltenyi Biotec), where the fraction was first treated with MACS Miltenyi kit CD303(BDCA-2) to isolate CD123^+^, then the remaining supernatant was treated with MACS Miltenyi kit CD141 (BDCA-3) (Miltenyi Biotec GmbH, #130-090-509, #130-090-512, Bergisch Gladbach, Germany) to isolate CD141^+^ DCs. The third tube (20°C) was used to isolate B cells with EasySep Human CD19 Positive Selection Kit (STEMCELL Technologies #18054 Vancouver, Canada). The markers chosen to isolate the APCs in this study (EpCAM, CD123, CD141 and CD19) are well established markers for human APCs, and have also previously been found in the respective human thymic APCs (see S2 Table). The cells were stored in RNAprotect Cell Reagent (Qiagen #76526) at -80°C. RNA was extracted from all cell types with RNeasy Plus Micro Kit (Qiagen #74034, Hilde, Germany).

### RNA-seq preparation

Because of low RNA yields, 100 pg of RNA from each cell type was used for amplifying cDNA with SMART-Seq v4 Ultra Low Input RNA Kit for Sequencing (Clontech Laboratories, CA, US). 1 ng of the cDNA was further prepared with MicroPlex Library Preparation Kit v2 (Diagenode, Seraing, Belgium). RNA sequencing (125 bp paired end) was performed at the Norwegian Sequencing center (NSC) on Illumina HiSeq 2500 (Illumina, CA, US) with 4 samples per lane.

### Pre-processing, alignment and quantification of RNA sequencing data

Low quality reads and adapter sequence was trimmed using Trimmomatic (Version 0.33); options ILLUMINACLIP:TrueSeq3-PE-2.fa:2:30:10:8:TRUE LEADING:3 SLIDINGWINDOW:4:15 MINLEN:36 [38]. PhiX sequence was removed with BBMap (Version 35.14) [39]. The reads were aligned with STAR (Version 2.4.2a) provided with both a reference genome (Homo_sapiens.GRCh38.dna_sm.primary_assembly.fa) and annotated transcripts (Homo_sapiens.GRCh38.80.gtf) from Ensembl, and options to decrease false positives by filtering out non-canonical unannotated and spurious junctions [40]. Duplicate aligned reads were removed with Picard Tools (Version 1.119), MarkDuplicates. Mapped reads were quantified using featureCounts (subread version 1.4.6-p3) summarizing paired-end reads, on both gene and exon level [41].

### Data processing in edgeR

The two datasets, quantified on gene level and exon level respectively, were further processed using the Bioconductor package edgeR [42] (Version 3.3.3). Tags expressed with less than 1 count-per-million (CPM) and 0.1 CPM and present in less than five biological replicates were filtered from the datasets quantified on gene level and exon level, respectively. Normalization was performed by using the edgeR calcNormFactors function. This function finds a a set of scaling factors for the library sizes that minimizes the log-fold changes between the samples for most genes [43]. To compute these scale factors, edgeR uses a trimmed mean of M-values (TMM) between each pair of samples.

After these processing steps, our APC dataset comprised 15245 Ensembl genes, where transcript levels were quantified in Fragments per Kilobase of transcript per Million mapped reads (FPKM). For all analyses, except the differential expression, differential splicing and GO enrichment analyses, median gene expression levels have been used.

### Quality control after purification of primary thymic APCs

An MDS plot for the 23 samples (five biological replicates for the mTECs, respectively, and six replicates for the CD19^+^ B cells, CD123^+^ and CD141^+^ DCs) was made in edgeR, based on the gene level quantifications from FeatureCounts. The two axes in the MDS plot correspond to the leading log_2_-fold change between each pair of samples. Leading log_2_-fold change is the root-mean square average of the largest log_2_-fold changes between each pair of samples. A venn diagram of the four APCs was made with the R package VennDiagram [44].

### Differential expression and GO enrichment analysis

Differential expression analysis was carried out in edgeR using generalized linear models (GLM) and GLM likelihood ratio tests to determine DE genes (log_2_ FC > 1 and FDR of < 0.05) between the cell types trough pairwise comparisons [42]. Differential exon usage analysis was performed by applying the edgeR F-test. A GO enrichment analysis was performed with all significant DE genes (log_2_ FC > 1 and FDR < 0.05) in the GOrilla software [45, 46](http://cbl-gorilla.cs.technion.ac.il/) using two unranked lists of genes (target and background lists). Only the pairwise comparisons involving mTECs obtained enough DE genes to return GO results. In order to reduce the figure sizes, the P-value threshold was set to 10^−9^ when DE genes in either CD141^+^, CD123^+^ or CD19^+^ cells were used as target list, whereas a P-value threshold of 10^−6^ was sufficient when DE genes in mTECs were used as target list.

### Genes encoding HLA and HLA pathway proteins

We examined the gene expression levels of the classical HLA genes (*HLA-A*, *HLA-B*, *HLA-C*, *HLA-DPA1*, *HLA-DPB1*, *HLA-DQA1*, *HLA-DQA2*, *HLA-DQB1*, *HLA-DQB2*, *HLA-DRA1* and *HLA-DRB1*), and genes involved in the HLA class I and class II pathways (*B2M*, *CALR*, *CANX*, *CD74*, *CTSB*, *CTSF*, *CTSS*, *ERAP1*, *ERAP2*, *HLA-DMA*, *HLA-DMB*, *HLA-DOA*, *HLA-DOB*, *IFI30*, *LGMN*, *PDIA3*, *PSMB5*, *PSMB8*, *TAP1*, *TAP2* and *TAPBP*) in the different APCs. Box plots were made with the R package ggplot2 [47].

### Transcriptional regulator genes, tissue enriched genes and genes encoding autoantigens in the thymic APCs

Boxplot of *AIRE*, *FEZF2* and *DEAF1* was made with the R package ggplot2. TRA genes were obtained from the HPA (Version 18) [26] (www.proteinatlas.org), where we downloaded the list of 2608 tissue-enriched genes from the tissue specific proteome. Genes annotated as “Tissue enriched” implicates that mRNA levels for these genes have been found to be at least five-fold higher in a particular tissue as compared to all other tissues in the database (data available from v16.proteinatlas.org). This list was further merged with our gene expression dataset. Our gene expression dataset only includes genes that are present in at least five biological replicates, and after merging, we found 601 of the tissue enriched genes in the APC dataset. However, as it has been reported in the literature that each individual TRA is lowly expressed in only 1–3% of mTECs at any given time, we lowered the filtering criteria in edgeR for this analysis and included genes present in at least one biological replicate in the dataset. We then found 1362 tissue enriched genes from our gene expression dataset. Furthermore, we searched for autoantigens associated with autoimmune diseases in the Immune Epitope Database [48](www.iedb.org) by setting the parameter “Disease” to “Autoimmune Disease” and “Host” to “Humans”. Among the 1733 autoantigens, 1402 were encoded by human genes. The list of the 1402 autoantigen genes was further merged with the 1362 tissue enriched genes in our dataset where we found 117 tissue enriched genes that overlapped with autoantigen genes. The venn diagram in Fig 4 were made with edgeR package VennDiagram [44]. The plot of total gene number across different FPKM thresholds was made with the R package ggplot2.

### Autoimmune disease genes

The eQTL genes were chosen from S1 Table in [27]. The microarray probes for the 10 eGenes with suggested significance were quality controlled as described in [27]. We further searched for these genes in our RNA sequencing data in the four thymic APCs.

## Supporting information

S1 Fig Analysis of genes encoding APC markers in the mTECs and the B cells. Genes encoding protein markers in (A) mTECs (EpCAM, FOXN1 and AIRE) and in (B) CD19^+^ B cells (CD19, CD22 and CD20 (MS4A1)).
(TIFF)

S2 Fig Analysis of genes encoding APC markers in the dendritic cell populations. Genes encoding protein markers in (A) CD123^+^ DCs (IL3RA (CD123), CLEC4C, NRP1 and LILRA4) and in (B) CD141^+^ DCs (CLEC9A, XCR1, THBD (CD141) and ITGAX (CD11c).
(TIFF)

S3 Fig Genes differentially expressed between the thymic APCs. MA plot displaying number of differentially expressed (DE) genes (log_2_ FC > 1) between (A) CD123^+^ (positive log_2_ FC) and CD141^+^ (negative log_2_ FC) (B) CD123^+^ (positive log_2_ FC) and CD19^+^ (negative log_2_ FC) (C) CD123^+^ (positive log_2_ FC) and mTEC (negative log_2_ FC) (D) CD141^+^ (positive log_2_ FC) and CD19^+^ (negative log_2_ FC) (E) CD141^+^ (positive log_2_ FC) and mTEC (negative log_2_ FC) and (F) CD19^+^ (positive log_2_ FC) and mTEC (negative log_2_ FC) under the generalized linear model (GLM) likelihood ratio test. The X-axis shows the average log_2_ count per million (CPM). The Y-axis shows the log_2_ fold change (FC) for each gene where positive and negative values are genes with higher expression levels in the first or the second cell type, respectively. The blue line represents the log_2_ fold change cut off (= 1) and red points are significant DE genes (FDR adjusted P-values < 0.05). The total number of significant DE genes is denoted in Table 1.
(TIFF)

S4 Fig GO analysis of significant DE genes (P < 0.05) in mTECs compared to CD141+ DCs. (TIFF)

S5 Fig GO analysis of significant DE genes (P < 0.05) in mTECs compared to CD123+ DCs. (TIFF)

S6 Fig GO analysis of significant DE genes (P < 0.05) in mTECs compared to CD19+ B cells. (TIFF)

S7 Fig GO analysis of significant DE genes (P < 0.05) in CD141+ DCs compared to mTECs. (TIFF)

S8 Fig GO analysis of significant DE genes (P < 0.05) in CD123+ DCs compared to mTECs. (TIFF)

S9 Fig GO analysis of significant DE genes (P < 0.05) in CD19+ B cells compared to mTECs. (TIFF)

S10 Fig Analysis of genes involved in the HLA class I pathway in the individual APC populations. (A) B2M (B) CALR (C) CANX (D) ERAP1 (E) ERAP2 (F) PDIA3 (Erp57) (G) PSMB5 (H) PSMB8 (I) TAP1 (J) TAP2 (K) TAPBP. Boxplots represent the median and quartiles of the relative RNA expression levels. The X-axis shows the individual thymic APCs and the Y-axis shows the TMM normalized FPKM. An expression level minimum has been set at FPKM = 1 (red line). Black dots represent the individual biological replicates. Black dots encircled in red are outliers.
(TIFF)

S11 Fig Analysis of genes involved in the HLA class II pathway in the individual APC populations. (A) *CD74* (B) *CTSB* (C) *CTSF* (D) *CTSS* (E) *HLA-DMA* (F) *HLA-DMB* (G) *HLA-DOA* (H) *HLA-DOB* (I) *IFI30* (J) *LGMN*. Boxplots represent the median and quartiles of the relative RNA expression levels. The X-axis shows the individual thymic APCs and the Y-axis shows the TMM normalized FPKM. An expression level minimum has been set at FPKM = 1 (red line). Black dots represent the individual biological replicates. Black dots encircled in red are outliers.
(TIFF)

S12 Fig Number of tissue enriched genes across different FPKM thresholds in the individual APCs. (TIFF)

S13 Fig Analysis of significant autoimmune disease associated eGenes in the individual APCs. (A) *FCRL3* (B) *ERAP2* (C) *RNASET2* (D) *SIRPG* (E) *SYS1*. Boxplots represent the median and quartiles of the relative RNA expression levels. The X-axis shows the individual thymic APCs and the Y-axis shows the TMM normalized FPKM. An expression level minimum has been set at FPKM = 1 (red line). Black dots represent the individual biological replicates. Black dots encircled in red are outliers.
(TIFF)

S14 Fig Analysis of autoimmune disease associated eGenes with suggestive significance (P-values < 7.4 x 10^−4^) in the individual APCs. (A) *AHI1*(B) *CLEC1* (C) *DDX59* (D) *ELMO1* (E) *GPR65* (F) *IP6K1* (G) *PARK7* (H) *SLC16A14* (I) *TRAIP* (J) *TROVE2*. Boxplots represent the median and quartiles of the relative RNA expression levels. The X-axis shows the individual thymic APCs and the Y-axis shows the TMM normalized FPKM. An expression level minimum has been set at FPKM = 1 (red line). Black dots represent the individual biological replicates. Black dots encircled in red are outliers.
(TIFF)

S1 Table Read numbers and percentages in the thymic APC samples during the preprocessing steps (mapping with STAR, search for duplicates with Picard (MarkDuplicates) and read assignment with featureCounts). (DOCX)

S2 Table List of proteins present in each human antigen presenting cell type and their respective genes used to validate the APC purity. (DOCX)

S3 Table List of uniquely and commonly expressed genes (FPKM > 1) in the four thymic APCs. (XLSX)

S4 Table List of significantly (FDR < 0.05) differentially expressed genes (log_2_ FC > 1) pairwise between the thymic APCs. (XLSX)

S5 Table DE (log_2_ fold change > 1 and FDR < 0.05) HLA genes between the thymic APCs. (DOCX)

S6 Table DE (log_2_ fold change > 1 and FDR < 0.05) HLA pathway genes between the thymic APCs. (DOCX)

S7 Table Expression levels (FPKM) of tissue-enriched genes (retrieved from the Human Protein Atlas) in the individual thymic APCs. (XLSX)

S8 Table Expression levels (FPKM) of tissue-enriched genes (retrieved from the Human Protein Atlas) that overlap with human autoantigen genes (retrieved from the Immune Epitope database) in the individual thymic APCs. (XLSX)
