# Supplementary figures and images for: Transcriptomes of antigen presenting cells in human thymus
Source: PLoS One. 2019 Jul 1;14(7):e0218858. doi: 10.1371/journal.pone.0218858 (PMC6602790; doi:10.1371/journal.pone.0218858)

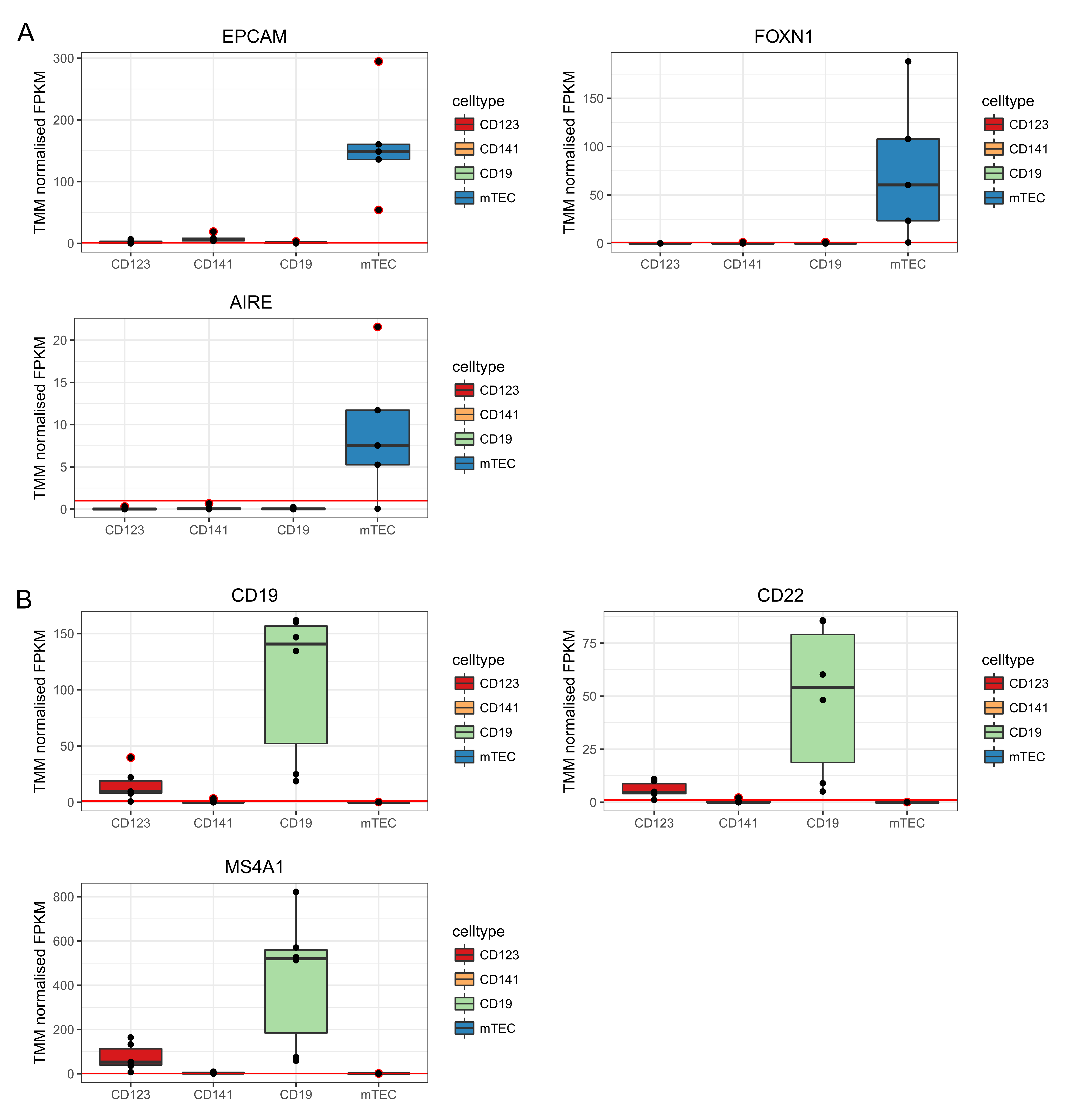

Supplement: S1 Fig — Genes encoding protein markers in (A) mTECs (EpCAM, FOXN1 and AIRE) and in (B) CD19+ B cells (CD19, CD22 and CD20 (MS4A1)). (TIFF) [file pone.0218858.s001.tiff]

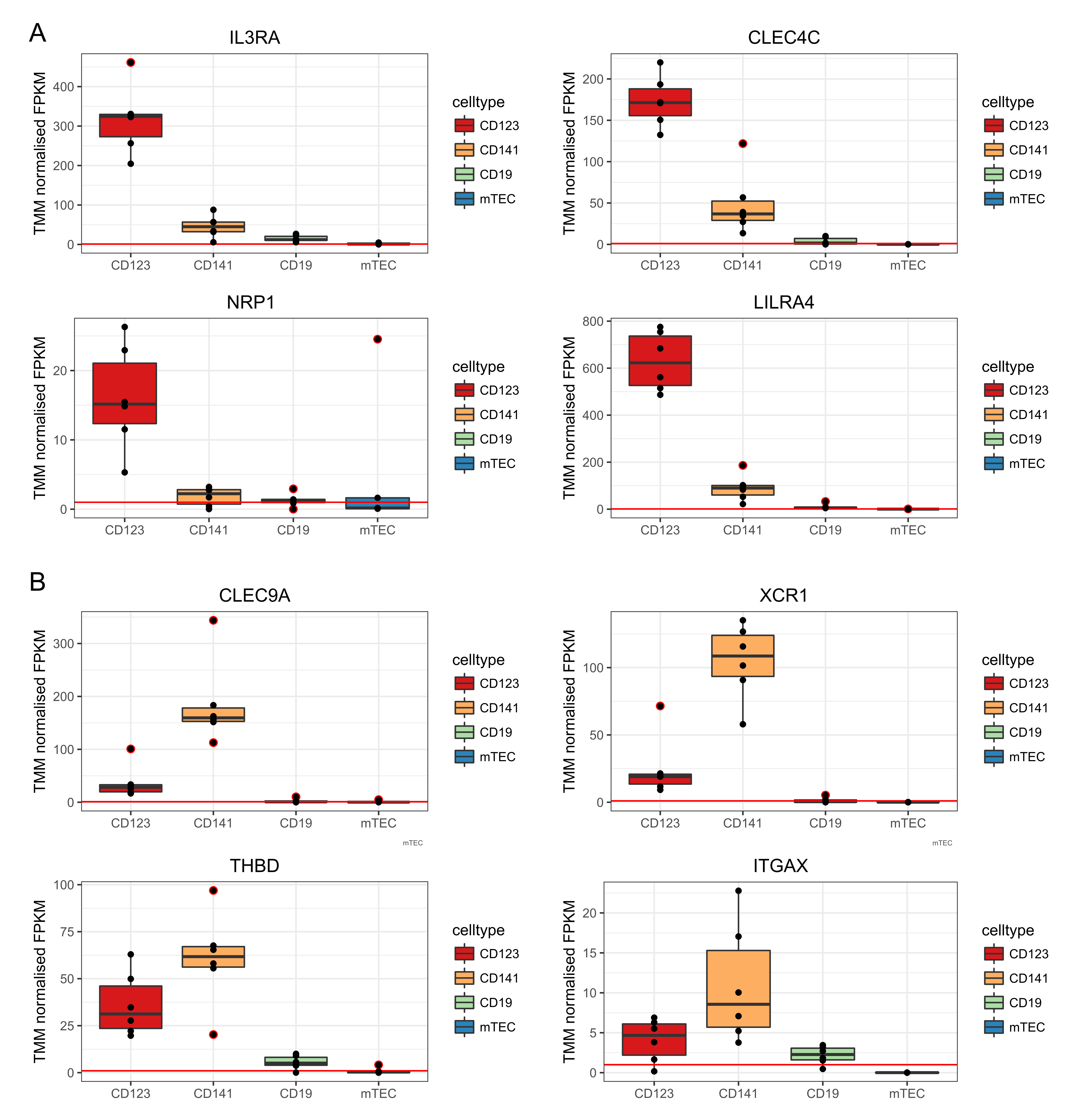

Supplement: S2 Fig — Genes encoding protein markers in (A) CD123+ DCs (IL3RA (CD123), CLEC4C, NRP1 and LILRA4) and in (B) CD141+ DCs (CLEC9A, XCR1, THBD (CD141) and ITGAX (CD11c). (TIFF) [file pone.0218858.s002.tiff]

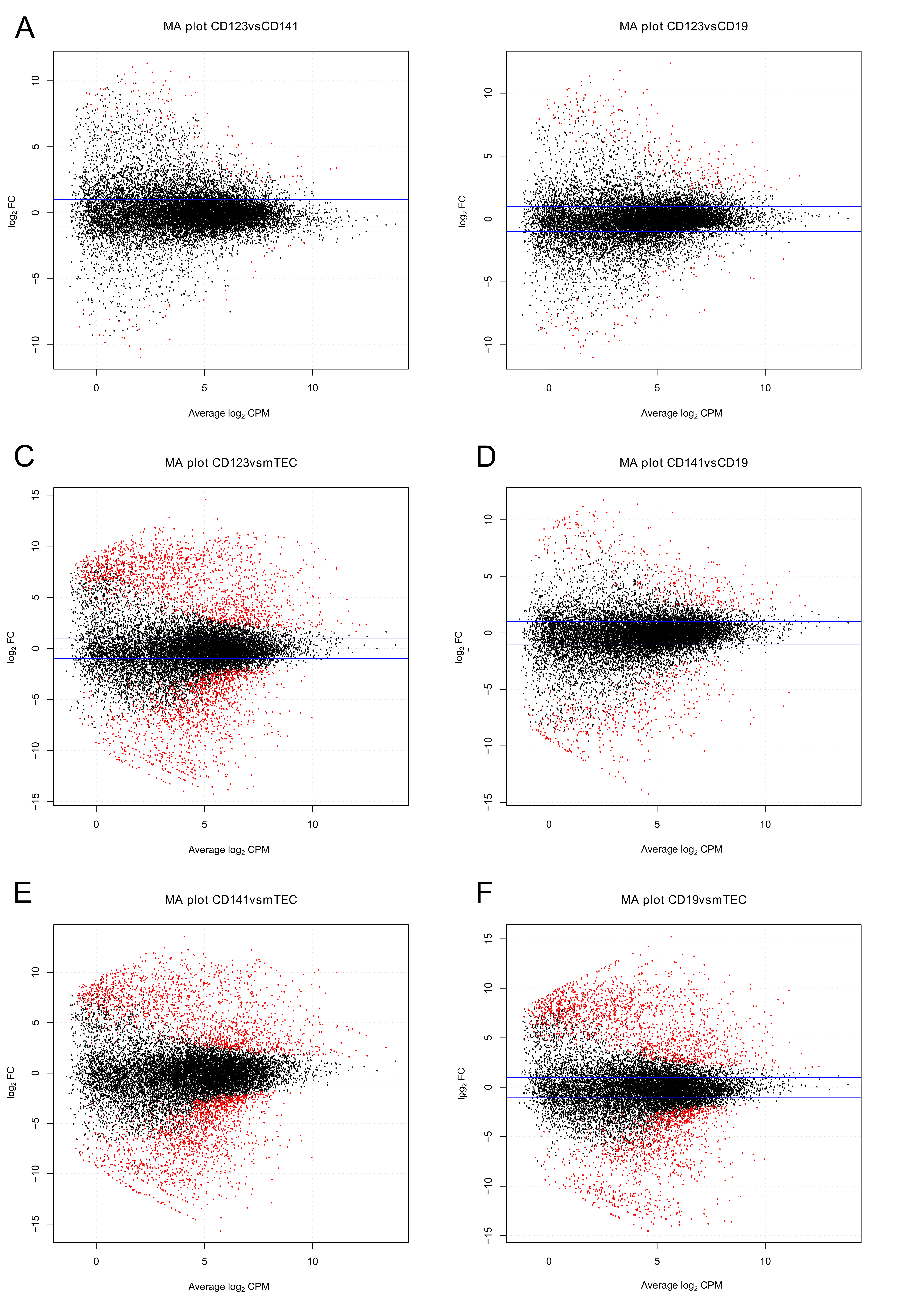

Supplement: S3 Fig — MA plot displaying number of differentially expressed (DE) genes (log2 FC > 1) between (A) CD123+ (positive log2 FC) and CD141+ (negative log2 FC) (B) CD123+ (positive log2 FC) and CD19+ (negative log2 FC) (C) CD123+ (positive log2 FC) and mTEC (negative log2 FC) (D) CD141+ (positive log2 FC) and CD19+ (negative log2 FC) (E) CD141+ (positive log2 FC) and mTEC (negative log2 FC) and (F) CD19+ (positive log2 FC) and mTEC (negative log2 FC) under the generalized linear model (GLM) likelihood ratio test. The X-axis shows the average log2 count per million (CPM). The Y-axis shows the log2 fold change (FC) for each gene where positive and negative values are genes with higher expression levels in the first or the second cell type, respectively. The blue line represents the log2 fold change cut off (= 1) and red points are significant DE genes (FDR adjusted P-values < 0.05). The total number of significant DE genes is denoted in Table 1. (TIFF) [file pone.0218858.s003.tiff]

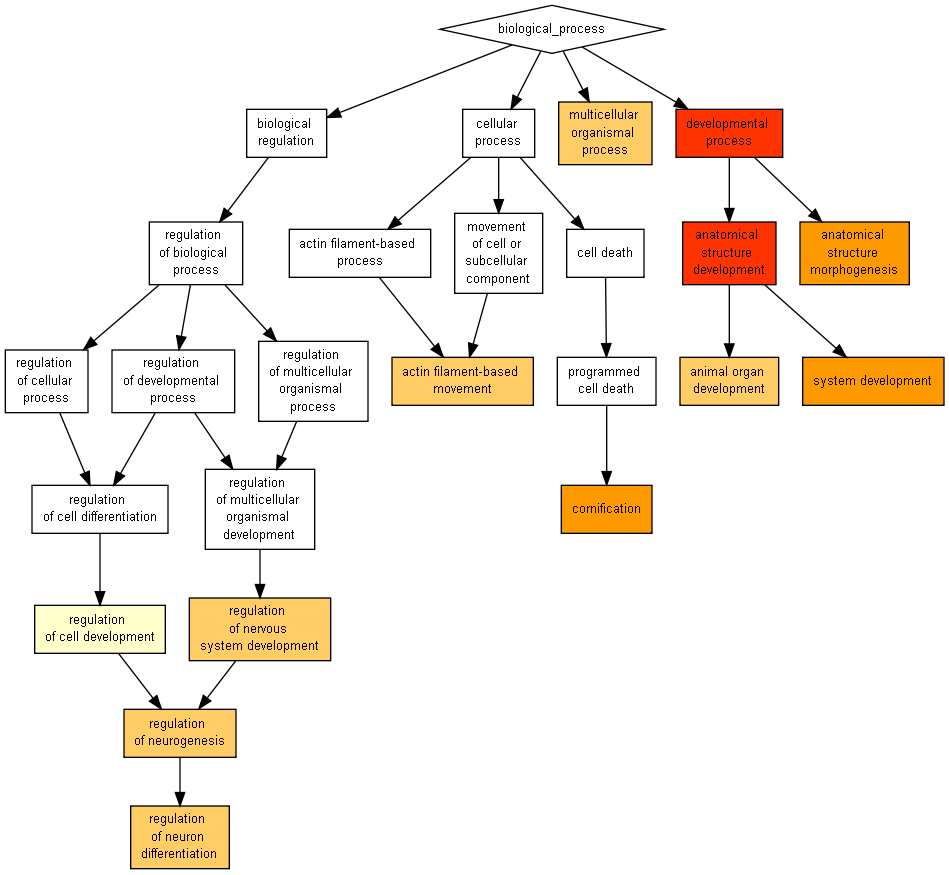

Supplement: S4 Fig — (TIFF) [file pone.0218858.s004.tiff]

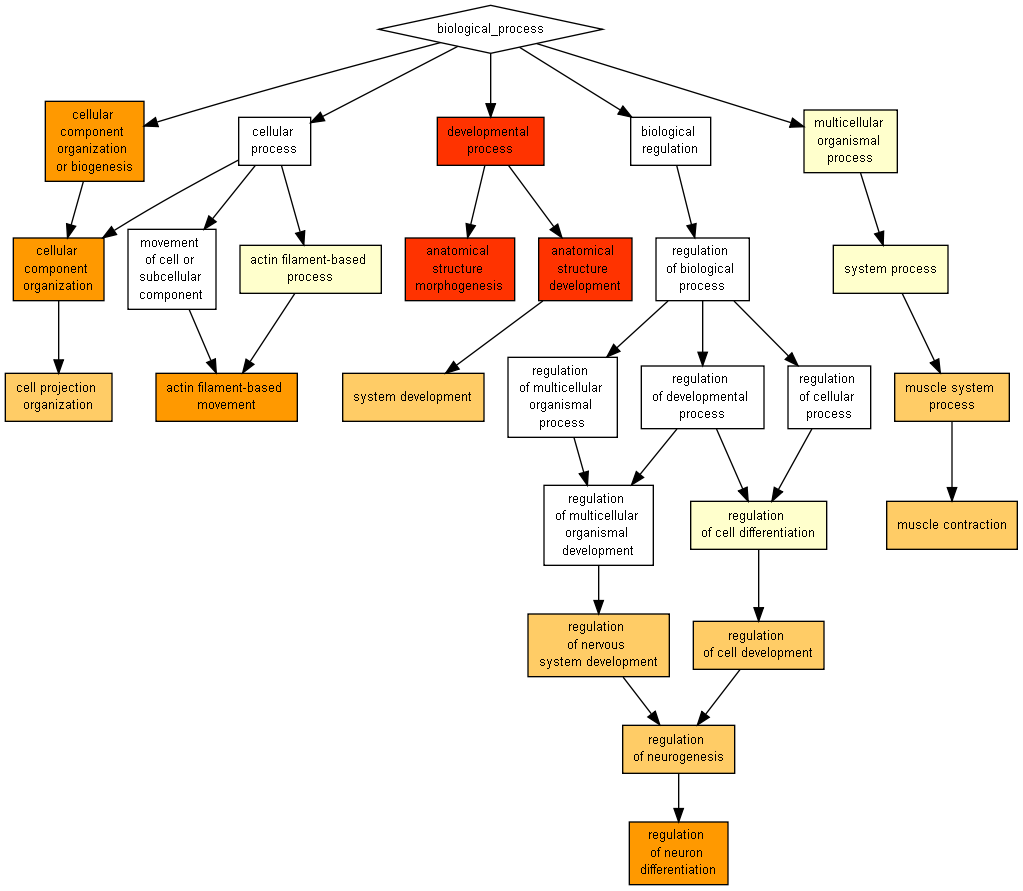

Supplement: S5 Fig — (TIFF) [file pone.0218858.s005.tiff]

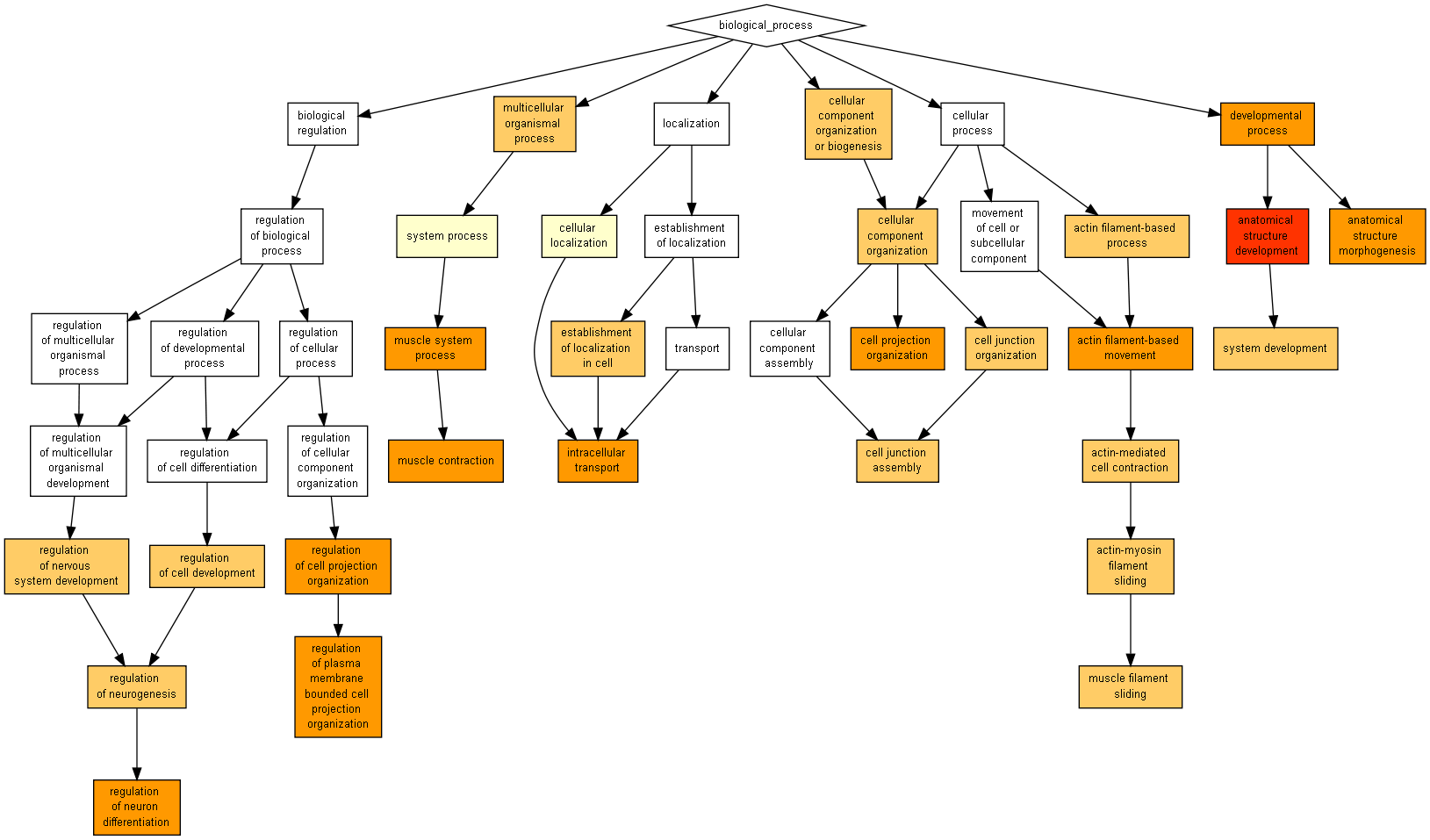

Supplement: S6 Fig — (TIFF) [file pone.0218858.s006.tiff]

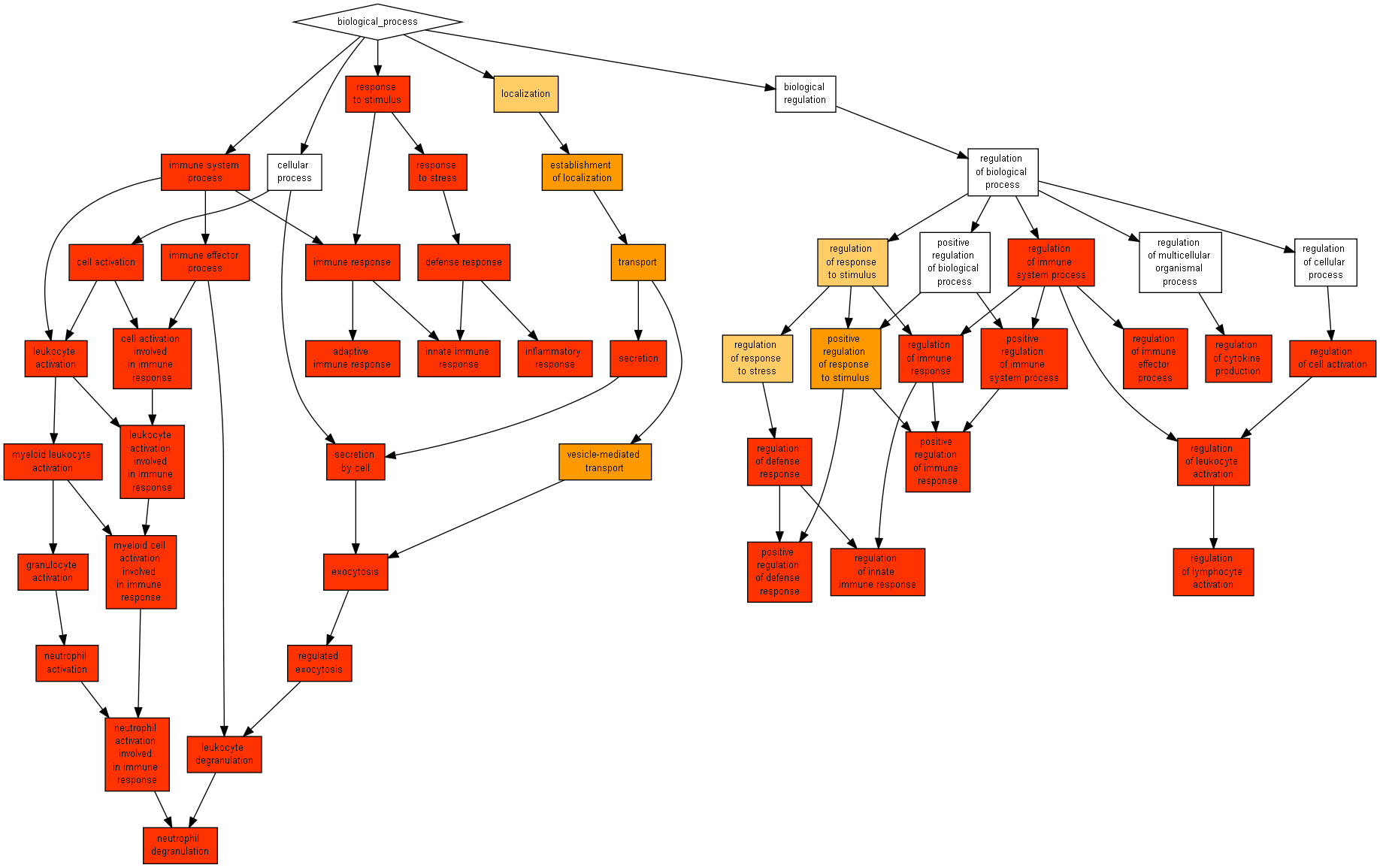

Supplement: S7 Fig — (TIFF) [file pone.0218858.s007.tiff]

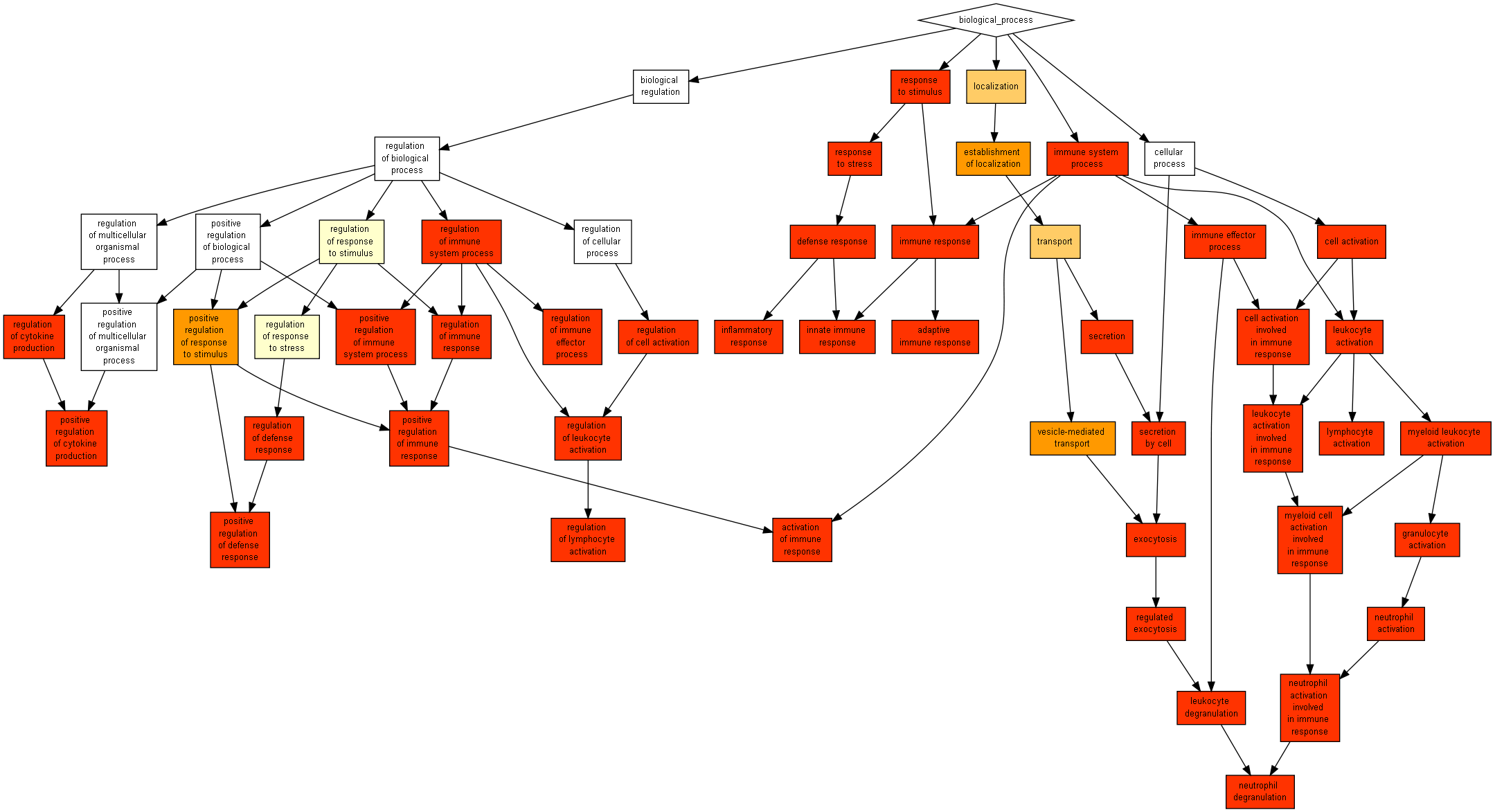

Supplement: S8 Fig — (TIFF) [file pone.0218858.s008.tiff]

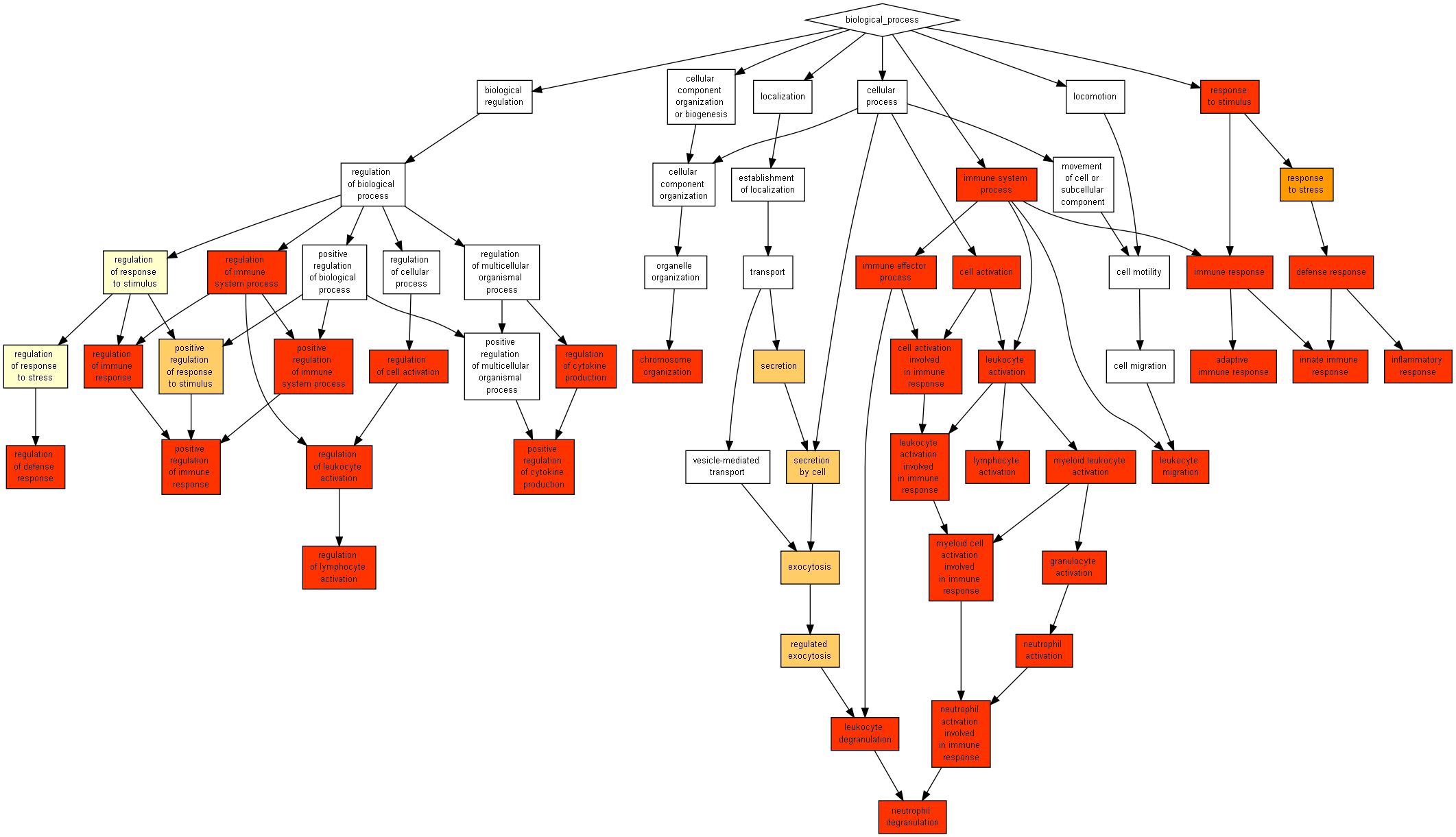

Supplement: S9 Fig — (TIFF) [file pone.0218858.s009.tiff]

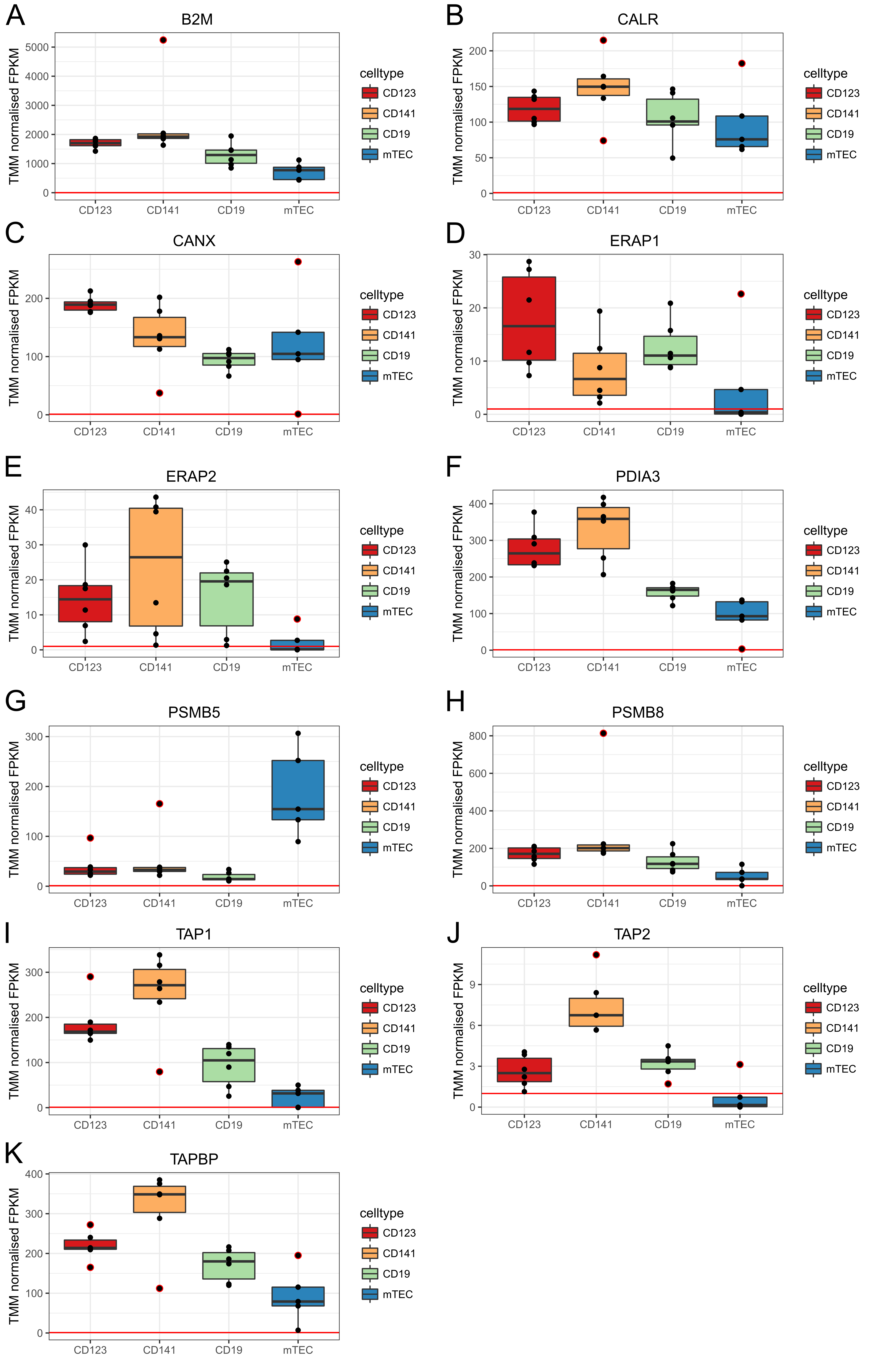

Supplement: S10 Fig — (A) B2M (B) CALR (C) CANX (D) ERAP1 (E) ERAP2 (F) PDIA3 (Erp57) (G) PSMB5 (H) PSMB8 (I) TAP1 (J) TAP2 (K) TAPBP. Boxplots represent the median and quartiles of the relative RNA expression levels. The X-axis shows the individual thymic APCs and the Y-axis shows the TMM normalized FPKM. An expression level minimum has been set at FPKM = 1 (red line). Black dots represent the individual biological replicates. Black dots encircled in red are outliers. (TIFF) [file pone.0218858.s010.tiff]

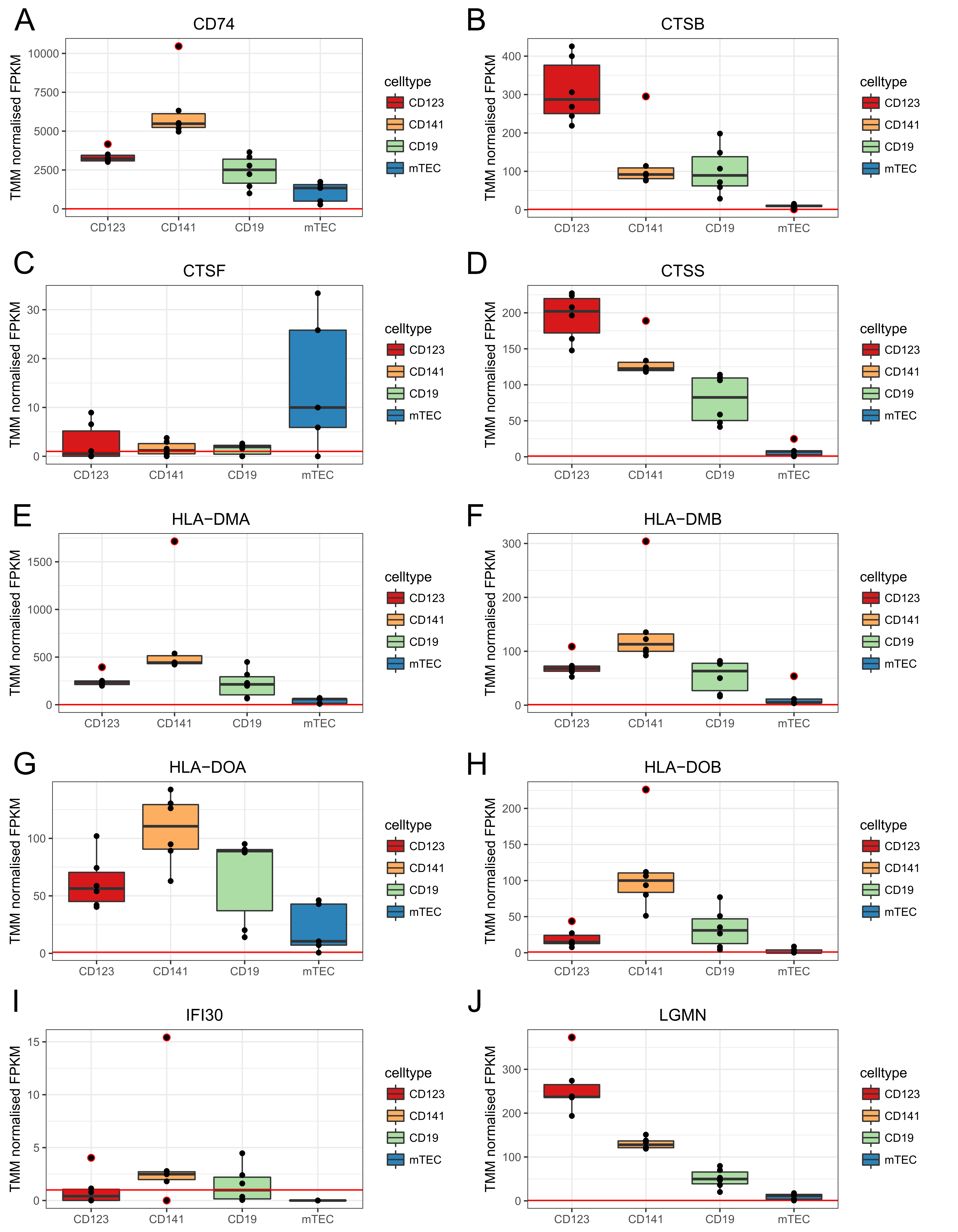

Supplement: S11 Fig — (A) CD74 (B) CTSB (C) CTSF (D) CTSS (E) HLA-DMA (F) HLA-DMB (G) HLA-DOA (H) HLA-DOB (I) IFI30 (J) LGMN. Boxplots represent the median and quartiles of the relative RNA expression levels. The X-axis shows the individual thymic APCs and the Y-axis shows the TMM normalized FPKM. An expression level minimum has been set at FPKM = 1 (red line). Black dots represent the individual biological replicates. Black dots encircled in red are outliers. (TIFF) [file pone.0218858.s011.tiff]

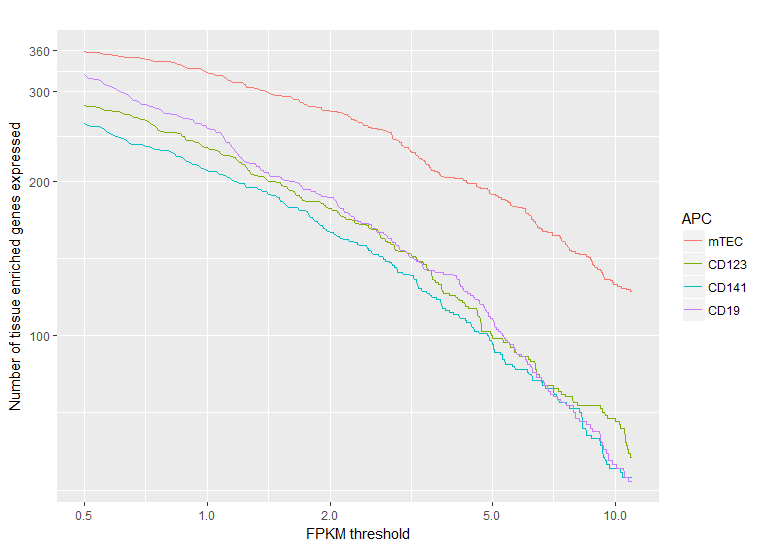

Supplement: S12 Fig — (TIFF) [file pone.0218858.s012.tiff]

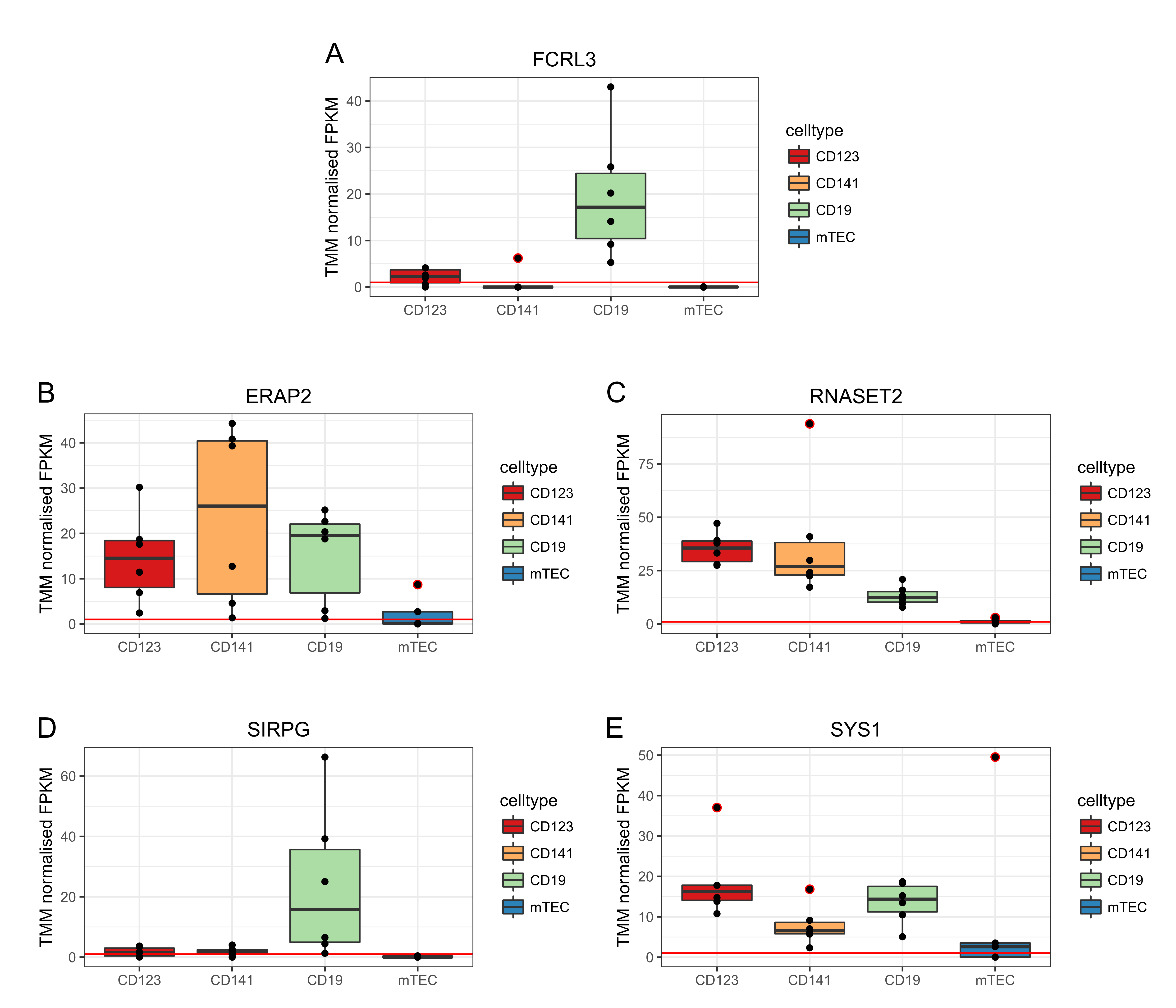

Supplement: S13 Fig — (A) FCRL3 (B) ERAP2 (C) RNASET2 (D) SIRPG (E) SYS1. Boxplots represent the median and quartiles of the relative RNA expression levels. The X-axis shows the individual thymic APCs and the Y-axis shows the TMM normalized FPKM. An expression level minimum has been set at FPKM = 1 (red line). Black dots represent the individual biological replicates. Black dots encircled in red are outliers. (TIFF) [file pone.0218858.s013.tiff]

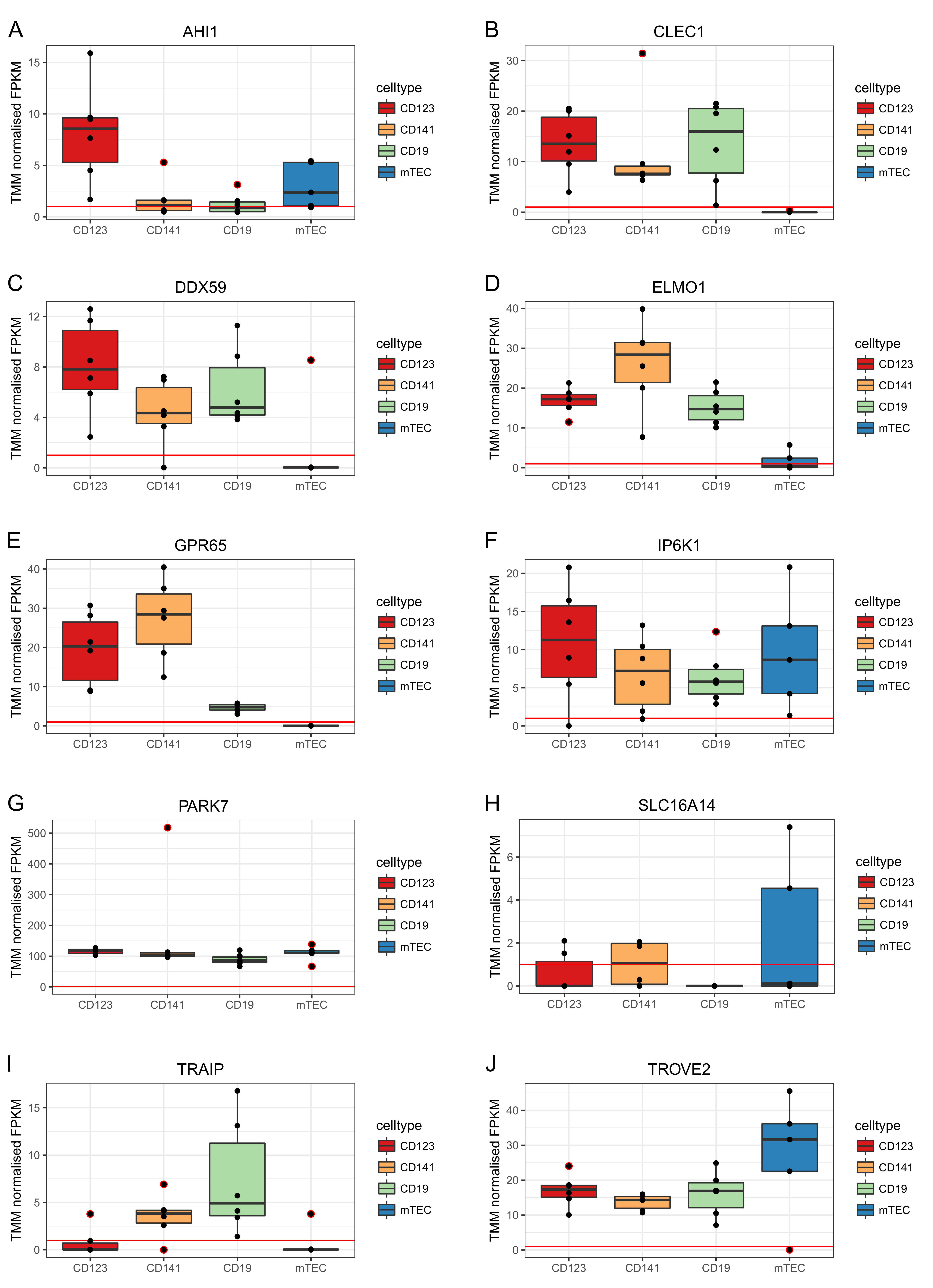

Supplement: S14 Fig — (A) AHI1(B) CLEC1 (C) DDX59 (D) ELMO1 (E) GPR65 (F) IP6K1 (G) PARK7 (H) SLC16A14 (I) TRAIP (J) TROVE2. Boxplots represent the median and quartiles of the relative RNA expression levels. The X-axis shows the individual thymic APCs and the Y-axis shows the TMM normalized FPKM. An expression level minimum has been set at FPKM = 1 (red line). Black dots represent the individual biological replicates. Black dots encircled in red are outliers. (TIFF) [file pone.0218858.s014.tiff]
