# Supplementary material for: Transcriptomes of antigen presenting cells in human thymus
Source: PLoS One. 2019 Jul 1;14(7):e0218858. doi: 10.1371/journal.pone.0218858 (PMC6602790; doi:10.1371/journal.pone.0218858)
Supplement: S1 Table — (DOCX) [file pone.0218858.s015.docx]

**Table S1.** **Read numbers and percentages in the thymic APC samples during the preprocessing steps (mapping with STAR, search for duplicates with Picard (MarkDuplicates) and read assignment with featureCounts).**

| Sample | STAR metrics | | MarkDuplicates metrics | featureCounts |
| --- | --- | --- | --- | --- |
|  | Total number of input (PE) reads | Uniquely mapped (PE) reads % | Percentage duplication of reads examined* | Successfully assigned fragments (percentage of total fragments) |
| 81-mTEC | 53602698 | 72.00 % | 78.57 % | 7219276 (68.1%) |
| 87-CD141 | 49705162 | 92.45 % | 56.03 % | 17574945 (71.6%) |
| 85-mTEC | 48508943 | 94.20 % | 45.90 % | 21200629 (75.8%) |
| 84-CD141 | 51803119 | 94.69 % | 44.62 % | 24276379 (77.6%) |
| 87-mTEC | 73147844 | 93.39 % | 59.21 % | 24354752 (75.5%) |
| 82-CD141 | 55559678 | 94.42 % | 45.39 % | 24709713 (73.7%) |
| 83-CD141 | 46692592 | 95.01 % | 37.31 % | 24809409 (80.4%) |
| 87-CD19 | 67951804 | 83.25 % | 48.28 % | 25168216 (63.6%) |
| 84-mTEC | 54719109 | 95.15 % | 42.49 % | 25234750 (75.7%) |
| 81-CD19 | 54721340 | 94.59 % | 43.13 % | 25953669 (74.8%) |
| 81-CD141 | 58416283 | 95.23 % | 46.66 % | 26624767 (78.6%) |
| 82-mTEC | 66011580 | 92.48 % | 49.18 % | 26677612 (74.4%) |
| 82-CD123 | 54850173 | 94.04 % | 36.49 % | 27383033 (71.2%) |
| 83-CD19 | 78181486 | 95.30 % | 57.07 % | 27574132 (71.7%) |
| 85-CD123 | 52790474 | 95.85 % | 33.82 % | 27943202 (76.3%) |
| 83-CD123 | 56589322 | 95.02 % | 39.21 % | 28798160 (77.3%) |
| 84-CD123 | 56622616 | 95.32 % | 38.59 % | 29158126 (79.1%) |
| 81-CD123 | 63755094 | 95.08 % | 43.40 % | 29803150 (76.3%) |
| 87-CD123 | 68448531 | 94.93 % | 46.60 % | 30566294 (77.1%) |
| 85-CD141 | 58089342 | 95.81 % | 38.14 % | 30919157 (81.8%) |
| 85-CD19 | 64362466 | 94.08 % | 40.99 % | 31151714 (75.9%) |
| 82-CD19 | 69927609 | 94.74 % | 36.13 % | 34791020 (72.3%) |
| 84-CD19 | 63129168 | 94.92 % | 30.38 % | 36600929 (77.6%) |

*The Picard tool MarkDuplicates examines both unpaired and paired reads
