# Supplementary material for: Transcriptomes of antigen presenting cells in human thymus
Source: PLoS One. 2019 Jul 1;14(7):e0218858. doi: 10.1371/journal.pone.0218858 (PMC6602790; doi:10.1371/journal.pone.0218858)
Supplement: S2 Table — (DOCX) [file pone.0218858.s016.docx]

Table S2: List of proteins present in each human antigen presenting cell type and their respective genes used to validate the APC purity

| Cell Type | Protein | Reference | Corresponding gene |
| --- | --- | --- | --- |
| mTEC | EpCAM | [1] | *EpCAM* |
|  | FOXN1 | [2] | *FOXN1* |
|  | AIRE | [3] | *AIRE* |
| CD19+ B cell | CD19 | [4] | *CD19* |
|  | CD22 | [5] | *CD22* |
|  | CD20 | [6] | *MS4A1* |
| CD123+ DC | CD123 | [7, 8] | *IL3RA* |
|  | CD303 | [7] | *CLEC4C* |
|  | CD304 | [7] | *NRP1* |
|  | CD85g (ILT7) | [8] | *LILRA4* |
| CD141+ DC | CLEC9A | [7, 8] | *CLEC9A* |
|  | XCR1 | [7] | *XCR1* |
|  | CD141 | [7, 8] | *THBD* |
|  | CD11c | [9] | *ITGAX* |

1. Trzpis M, McLaughlin PM, de Leij LM, Harmsen MC. Epithelial cell adhesion molecule: more than a carcinoma marker and adhesion molecule. Am J Pathol. 2007;171(2):386-95. doi: 10.2353/ajpath.2007.070152. PubMed PMID: 17600130; PubMed Central PMCID: PMCPMC1934518.

2. Romano R, Palamaro L, Fusco A, Giardino G, Gallo V, Del Vecchio L, et al. FOXN1: A Master Regulator Gene of Thymic Epithelial Development Program. Front Immunol. 2013;4:187. doi: 10.3389/fimmu.2013.00187. PubMed PMID: 23874334; PubMed Central PMCID: PMCPMC3709140.

3. Peterson P, Org T, Rebane A. Transcriptional regulation by AIRE: molecular mechanisms of central tolerance. Nature reviews Immunology. 2008;8(12):948-57. doi: 10.1038/nri2450. PubMed PMID: 19008896; PubMed Central PMCID: PMCPMC2785478.

4. Wang K, Wei G, Liu D. CD19: a biomarker for B cell development, lymphoma diagnosis and therapy. Exp Hematol Oncol. 2012;1(1):36. doi: 10.1186/2162-3619-1-36. PubMed PMID: 23210908; PubMed Central PMCID: PMCPMC3520838.

5. Ereno-Orbea J, Sicard T, Cui H, Mazhab-Jafari MT, Benlekbir S, Guarne A, et al. Molecular basis of human CD22 function and therapeutic targeting. Nature communications. 2017;8(1):764. doi: 10.1038/s41467-017-00836-6. PubMed PMID: 28970495; PubMed Central PMCID: PMCPMC5624926.

6. Kehrl JH, Riva A, Wilson GL, Thevenin C. Molecular mechanisms regulating CD19, CD20 and CD22 gene expression. Immunol Today. 1994;15(9):432-6. doi: 10.1016/0167-5699(94)90273-9. PubMed PMID: 7524520.

7. Schlitzer A, Ginhoux F. Organization of the mouse and human DC network. Curr Opin Immunol. 2014;26:90-9. doi: 10.1016/j.coi.2013.11.002. PubMed PMID: 24556405.

8. Gurka S, Dirks S, Photiadis J, Kroczek RA. Expression analysis of surface molecules on human thymic dendritic cells with the 10th HLDA Workshop antibody panel. Clin Transl Immunology. 2015;4(10):e47. doi: 10.1038/cti.2015.21. PubMed PMID: 26682055; PubMed Central PMCID: PMCPMC4673441.

9. Vandenabeele S, Hochrein H, Mavaddat N, Winkel K, Shortman K. Human thymus contains 2 distinct dendritic cell populations. Blood. 2001;97(6):1733-41. PubMed PMID: 11238115.
