# Supplementary material for: Transcriptomes of antigen presenting cells in human thymus
Source: PLoS One. 2019 Jul 1;14(7):e0218858. doi: 10.1371/journal.pone.0218858 (PMC6602790; doi:10.1371/journal.pone.0218858)
Supplement: S5 Table — (DOCX) [file pone.0218858.s019.docx]

**Table S5.** DE (log fold change > 1 and FDR < 0.05) HLA genes between the thymic APCs

| Cell Type 1 | Cell Type 2 | HLA genes with significantly higher expression in Cell Type 1 | FDR P-value |
| --- | --- | --- | --- |
| CD141^+^ | mTEC | HLA-DPA1 | 1.85E-11 |
|  |  | HLA-DPB1 | 5.19E-11 |
|  |  | HLA-DRB1 | 6.33E-09 |
|  |  | HLA-DQB1 | 1.24E-08 |
|  |  | HLA-DRA1 | 1.36E-08 |
|  |  | HLA-DQA1 | 1.79E-06 |
|  |  | HLA-B | 0.000178 |
|  |  | HLA-C | 0.005121 |
|  |  | HLA-A | 0.045168 |
| CD123^+^ | mTEC | HLA-DPB1 | 0.000665 |
|  |  | HLA-DPA1 | 0.001256 |
|  |  | HLA-DRA1 | 0.002498 |
|  |  | HLA-DQB1 | 0.00285 |
|  |  | HLA-DRB1 | 0.004051 |
|  |  | HLA-DQA1 | 0.017446 |
|  |  | HLA-B | 0.023396 |
| CD19^+^ | mTEC | HLA-DQB1 | 0.00394 |
|  |  | HLA-DRA1 | 0.009066 |
|  |  | HLA-DRB1 | 0.01659 |
|  |  | HLA-DPA1 | 0.024217 |
|  |  | HLA-DPB1 | 0.037065 |
|  |  | HLA-DQA1 | 0.040317 |
| CD141^+^ | CD19^+^ | HLA-DPB1 | 0.001269 |
|  |  | HLA-DPA1 | 0.003532 |
