# Supplementary material for: Transcriptomes of antigen presenting cells in human thymus
Source: PLoS One. 2019 Jul 1;14(7):e0218858. doi: 10.1371/journal.pone.0218858 (PMC6602790; doi:10.1371/journal.pone.0218858)
Supplement: S6 Table — (DOCX) [file pone.0218858.s020.docx]

**Table S6.** DE (log fold change > 1 and FDR < 0.05) HLA pathway genes between the thymic APCs

| Cell type 1 | Cell type 2 | HLA pathway gene with significantly higher gene expression in cell type 1 | FDR P-value |
| --- | --- | --- | --- |
| mTEC | CD123^+^ | PSMB5 | 0.025093 |
| mTEC | CD19^+^ | PSMB5 | 0.000121 |
| CD141^+^ | mTEC | LGMN | 5.26E-07 |
|  |  | CTSS | 5.38E-07 |
|  |  | HLA-DMA | 9.56E-07 |
|  |  | CTSB | 1.51E-06 |
|  |  | HLA-DOB | 2.17E-05 |
|  |  | CD74 | 8.11E-05 |
|  |  | IFI30 | 0.000331 |
|  |  | HLA-DMB | 0.000512 |
|  |  | TAP1 | 0.001332 |
|  |  | PSMB8 | 0.011384 |
|  |  | B2M | 0.018472 |
|  |  | TAP2 | 0.023799 |
|  |  | ERAP2 | 0.026382 |
|  |  | HLA-DOA | 0.028575 |
|  |  | PDIA3 | 0.041385 |
| CD123^+^ | mTEC | CTSB | 2.22E-11 |
|  |  | LGMN | 6.82E-11 |
|  |  | CTSS | 6.94E-09 |
|  |  | IFI30 | 0.002257 |
|  |  | HLA-DMA | 0.004402 |
|  |  | TAP1 | 0.006065 |
|  |  | HLA-DOB | 0.023917 |
|  |  | HLA-DMB | 0.03156 |
| CD123^+^ | CD19^+^ | LGMN | 0.029433 |
| CD19^+^ | mTEC | CTSB | 1.22E-05 |
|  |  | CTSS | 0.000105 |
|  |  | IFI30 | 0.00133 |
|  |  | HLA-DOB | 0.00366 |
|  |  | LGMN | 0.005438 |
|  |  | HLA-DMA | 0.010756 |
